# Diversification of the *Salmonella* Fimbriae: A Model of Macro- and Microevolution

**DOI:** 10.1371/journal.pone.0038596

**Published:** 2012-06-12

**Authors:** Min Yue, Shelley C. Rankin, Ryan T. Blanchet, James D. Nulton, Robert A. Edwards, Dieter M. Schifferli

**Affiliations:** 1 Department of Pathobiology, University of Pennsylvania School of Veterinary Medicine, Philadelphia, Pennsylvania, United States of America; 2 Department of Computer Science, College of Sciences, San Diego State University, San Diego, California, United States of America; 3 Mathematics and Computer Science Division, Argonne National Laboratory, Argonne, Illinois, United States of America; University of Osnabrueck, Germany

## Abstract

Bacteria of the genus *Salmonella* comprise a large and evolutionary related population of zoonotic pathogens that can infect mammals, including humans and domestic animals, birds, reptiles and amphibians. *Salmonella* carries a plethora of virulence genes, including fimbrial adhesins, some of them known to participate in mammalian or avian host colonization. Each type of fimbria has its structural subunit and biogenesis genes encoded by one fimbrial gene cluster (FGC). The accumulation of new genomic information offered a timely opportunity to better evaluate the number and types of FGCs in the *Salmonella* pangenome, to test the use of current classifications based on phylogeny, and to infer potential correlations between FGC evolution in various *Salmonella* serovars and host niches. This study focused on the FGCs of the currently deciphered 90 genomes and 60 plasmids of *Salmonella.* The analysis highlighted a fimbriome consisting of 35 different FGCs, of which 16 were new, each strain carrying between 5 and 14 FGCs. The *Salmonella* fimbriome was extremely diverse with FGC representatives in 8 out of 9 previously categorized fimbrial clades and subclades. Phylogenetic analysis of *Salmonella* suggested macroevolutionary shifts detectable by extensive FGC deletion and acquisition. In addition, microevolutionary drifts were best depicted by the high level of allelic variation in predicted or known adhesins, such as the type 1 fimbrial adhesin FimH for which 67 different natural alleles were identified in *S. enterica* subsp. I. Together with strain-specific collections of FGCs, allelic variation among adhesins attested to the pathoadaptive evolution of *Salmonella* towards specific hosts and tissues, potentially modulating host range, strain virulence, disease progression, and transmission efficiency. Further understanding of how each *Salmonella* strain utilizes its panel of FGCs and specific adhesin alleles for survival and infection will support the development of new approaches for the control of Salmonellosis.

## Introduction

*Salmonella* infections result in substantial human and livestock morbidity and mortality worldwide [1]. In humans *S. enterica* serovars Typhi and Paratyphi cause systemic diseases (typhoid and paratyphoid fever), globally with an estimated 12–33 million cases of illness and 216,00–600,000 deaths per year [2]. Non-typhoidal salmonellae cause foodborne diarrheal illness, with approximately 1.3 billion cases of gastroenteritis per year, resulting in 3 million deaths [3]. *Salmonella* remains the most frequent bacterial agent of foodborne diseases [4], [5] and was the leading foodborne microbe causing hospitalizations and deaths in the US [6]. *Salmonella* affects also animals, and immunologically unprepared young, stressed or periparturient farm animals are particularly susceptible to *Salmonella enterica* strains capable of causing systemic infections [7]–[12]. More frequently following an enteric infection, farm animals become asymptomatic carriers that shed bacteria contaminating carcasses, milk, eggs and agricultural products grown on land fertilized with manure [13]. Undetected animal reservoirs best explain why CDC surveillance programs aimed at reducing food contamination remain mostly unsuccessful for *Salmonella* [4], [14].

*Salmonella*e are thought to have diverged from a common ancestor with *Escherichia coli* 100∼160 million years ago [15]. Although the latest accepted nomenclature divides *Salmonella* in only two species, bongori and enterica, and the latter species in 6 main named or numbered subspecies (*enterica* or I, *salamae* or II, *arizonae* or IIIa, *diarizonae* or IIIb, *houtenae* or IV and *indica* or VI; V is now *S. bongori*) [16], over 2,600 serovars have been identified [17]. Serovars are defined by the antigenic properties of the polysaccharide chains of LPS (O-antigens) and of the proteinaceous flagella (H antigens). *Salmonella* inhabit and multiply in an environment that is highly propitious for horizontal gene transfer (HGT): the intestine of carrier animals which is extremely rich in mobile DNA. O- and H-antigen gene studies indicated that the acquisition of DNA played a major role in the diversification of the *Salmonella* serovar antigens [18], [19]. Newly acquired serovar-modifying DNA, together with the elimination or inactivation of unnecessary or interfering DNA, has been suggested to direct serovar-specific adaptation for successful competition with the host-specific intestinal flora, and provide the defense against predatory protozoa, lytic phages and host-specific immunity [20]–[22]. Diagnostic and epidemiological focus on the serovars of *Salmonella* has led to the distinction of serovars that are host-restricted (e.g. serovar Gallinarum in birds or Typhi in humans), host-adapted (e.g. serovar Choleraesuis in swine, more rarely in other animals or in humans), and broad range (e.g. serovar Typhimurium). However, the exact genetic components that determine host range and specialized adaptation remain to be identified.

A variety of methods have been used to dissect evolutionary links between serovars such as multi-locus enzyme electrophoresis [23] and multi-locus sequence typing (MLST), typically based on up to 7 housekeeping genes (http://mlst.ucc.ie/mlst/dbs/Senterica). The latter approach was able to demonstrate that not all *Salmonella* subspecies are clonal and detected inter-subspecies HGT events [24]. Use of MLST data for subspecies I highlighted clonal separations for several serovars [25] and suggested clonal adaptation by recombination mechanisms that occur independently of the O and H antigens. Comparative studies of MLST with microarrays that included some virulence genes attributed discordant phylogenic associations with serovars due to the stronger participation of HGT for virulence factors, which are frequently associated with mobile DNA elements [26]. The current study took advantage of the accumulation of genomic data to improve the accuracy of a phylogenomic analysis that proposes an evolutionary history of *Salmonella* [27].

Beside the O- and H-antigens, other surface-exposed components of *Salmonella* have been the targets of evolutionary adaptation to changing selective conditions of the environment. A range of variable strain-specific surface proteins has endowed *Salmonella* with the capability to colonize and adapt to different ecological niches and hosts [28], [29]. In humans and animals, *Salmonella* infections are acquired orally and start by productive interactions between bacterial and intestinal surface molecules. These early interactions are typically mediated by bacterial adhesins that act as initiators of intestinal surface colonization or as a prerequisite for local invasion and/or systemic spread [28], [30]. Several studies have illustrated the involvement of both fimbrial and non-fimbrial ligands or adhesins in the colonization of avian and mammalian intestines [31]–[33]. Most *Salmonella* fimbriae belong to the chaperone-usher group of fimbriae, best studied in *E. coli* [34], [35]. These fimbriae have one or more structural subunits that are exported and assembled in an ordered manner on the bacterial surface by cognate periplasmic chaperone protein(s) and an outer-membrane usher protein. The genes for each type of fimbria are grouped in one cluster, sometimes together with gene(s) involved in regulating fimbrial expression. Fimbrial structures are either homopolymeric or more frequently, heteropolymeric with an adhesive minor protein subunit at the fimbrial tip. Less frequently, the major subunit is the adhesin [36] or the fimbriae have more than one subunit with adhesive properties [37]–[39]. In contrast to the type IV pili, which are rarely found in livestock *S. enterica* serovars [40], most if not all *S. enterica* express curli, a fimbria-like structure that uses a different export apparatus than the large chaperone-usher group and that is involved in biofilm formation [41]–[43]. Moreover, *Salmonella* also carries genes for the expression of outer membrane proteins that expose surface domains with adhesive properties [44], [45]. Many of these non-polymeric adhesins are autotransporter proteins that export the N-terminal region (or passenger domain that includes the adhesive moiety) through a β-barrel membrane channel formed by their C-terminus [46]–[48].

*Salmonella* carries different types of chaperone-usher fimbriae, some of them known to be involved in binding to different receptors, persisting in specific niches, promoting infections or forming biofilms. While gene clusters for many different fimbrial adhesins are carried by all *Salmonella* serovars, some are restricted to a particular host [49], [50], suggesting a potential role for fimbriae in regulating host specificity. Distinct sets of fimbriae are involved in the differential intestinal colonization of animal species [51], [52] and participate in host adaptation [53]. Several studies have highlighted how one or a few amino acid substitutions in the mannose-inhibitable type 1 fimbrial adhesin FimH of *Salmonella* can modulate receptor-, host- or cell-type binding specificities [54]–[56] and affect the efficiency of uptake by professional phagocytes [57]. The orthologous FimH of the avian-specific serovars Gallinarum and Pullorum mediated significantly better bacterial binding to chicken leukocytes than serovar Typhimurium FimH alleles. This avian-specific FimH did not mediate bacterial binding to mammalian cells and binding to chicken leucocytes was only minimally inhibited by mannose, confirming that these adhesins recognized different receptors. FimH of serovar Gallinarum and Typhimurium differ by only 5–6 amino acids [58], highlighting how allelic variation of the *Salmonella* FimH adhesin directs not only host-cell-specific recognition, but also distinctive binding to mammalian or avian receptors. Remarkably, the allele-specific binding profile paralleled the host specificity of the respective FimH-expressing pathogen [59]. Based on these findings, it is most likely that the binding properties of individual bacterial strains are not just influenced by multiple adhesins, but also by unique sets of adhesin alleles. However, only a minority of adhesins have been identified and characterized experimentally in *Salmonella*. The accumulation of new information with the increasing number of sequenced *Salmonella* genomes compels a reevaluation of the number and distribution of fimbrial gene clusters (FGCs) in this genus. Here, we propose to analyze the features of the *Salmonella* fimbriome (the collection of fimbrial types in the *Salmonella* pangenome) by using the currently available genomes.

This study takes advantage of the availability of 90 fully sequenced *Salmonella* genomes and 60 sequenced plasmids to catalogue a list of *Salmonella*-specific FGCs, each defined as one functional unit of co-evolved genes. To describe and classify all of the FGCs detected, phylogenetic analysis was used to determine whether the evolution of the *Salmonella* fimbriome is associated with the evolutionary history of *Salmonella*. The data from this study indicate that extensive acquisition and loss of FGCs led to different lineages with distinct pathogenic capabilities. The allelic variation detected within most known or predicted fimbrial adhesins supports previous studies that highlighted the adaptation of *Salmonella* towards host- and tissue-specificity, potentially modulating strain virulence, disease progression, and transmission efficiency.

## Results and Discussion

### Core and Accessory FGCs of the *Salmonella* Fimbriome

**Figure 1:**
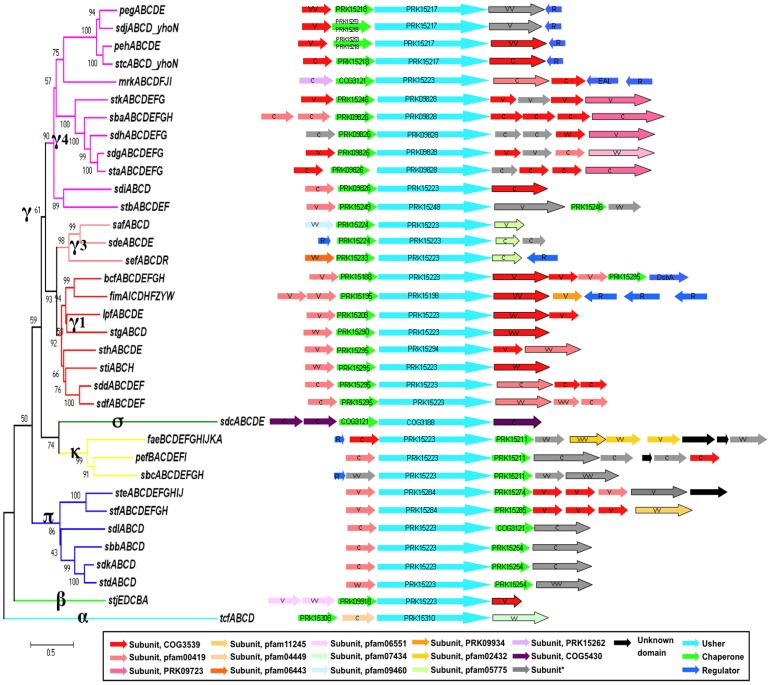
Chaperone-usher fimbrial gene clusters (FGCs) of *Salmonella*. A phylogenetic tree was built for 35 types of FGCs by using the amino acid sequences of the combined 950 usher proteins from 90 genomes (MEGA 5.0, as described in method). The FGCs were divided into five clades. The scale indicates the number of substitutions per amino acid. The bottom box lists different protein domain families. The asterisk indicates that some subunits were not picked by CDD or InterPro Scan, but (i) showed sequence similarity with other subunit(s) in the same gene clusters and (ii) were typically β-sheet-rich, as are all fimbrial subunits. Framed arrows are either known or predicted adhesins (as described in the text). C, V, VV, VVV were used to define the level of amino sequence variability for each subunit. C indicates subunits for which there was only one sequence available, or subunits lacking variants; V, VV or VVV indicated respectively ≤1, 1–10 or >10 detected variations per 100 amino acids.

Whereas only 15 *Salmonella* usher proteins were described in 2007 [34], the current study detected 35 unique types of usher in April 2011 by taking advantage of 90 available *Salmonella* genomes (27 distinct serovars). All the ORFs encoding fimbrial usher proteins had neighboring ORFs for chaperone proteins and two or more fimbrial subunits, as recognized by protein sequence similarities to known fimbrial subunits. Interestingly, there were no orphan ushers for a total of 950 ushers (with ≥85% amino acid sequence identities), and 35 different types of fimbrial gene clusters as shown in Figure 1. The 35 FGCs were clearly different from each other (<35% amino acid identity) and only 22 were previously named. On average, *Salmonella* were found to have 11.8 FGCs per strain with over 85% of the strains or serovars having between 11 and 13 FGCs (Figure S1). Notably, *S. enterica* subspecies *arizonae* had the least number of FGCs, (only 5), and *S. bongori* and *S. enterica* subspecies *diarizonae* both had 8 FGCs. Subspecies *enterica* serovar Pullorum and Weltevreden had 10 FGCs, whereas serovars Virchow and Kentucky carried the most FGCs with 14. Since the collection of 90 *Salmonella* strains studied (i) doesn’t include all the known (and unknown) serovars, and (ii) is skewed by having over- and underrepresented serovars, subspecies and species (most being *S. enterica* subsp. I), models evaluating the number of different FGCs in the *Salmonella* pangenome remain too speculative. However, a model based on the 26 FGCs from strains of serovars that had at least two different complements of FGCs predicted that the pangenome of *S. enterica* subsp. I has 42 to 43 different FGCs (Figure S2).

To address evolutionary questions about all the 35 individual FGCs, it was necessary to determine which FGCs were present in each *Salmonella* strain. For this, a phylogenomic tree based on 45 highly conserved housekeeping genes of the 90 sequenced *Salmonella* strains was compared to the panel of FGCs present in each strain (Figure S3). This tree classifies the serovars in five phylogenic clades numbered 1 to 5. Clade 5 represents *Salmonella* serovars that characteristically associate with cold-blooded animals. The analysis established that the *bcf, fim, stb, sth, std, saf* and *sti* FGCs were present in most *Salmonella* strains and serovars (>80% for both), albeit as pseudogenes in some serovars (core FGCs, Figure S4). The *bcf* FGC was the only one that was conserved in all *Salmonella* strains. The predominance of these FGCs suggests that they exert some important or even essential function for *Salmonella* survival, such as colonization, virulence and/or transmission. The absence of some of these core FGCs seemed to be partially serovar-specific, such as the association of *std* with serovar Pullorum and Gallinarum. However, such observations need to be confirmed with higher numbers of strains per serovar. The *saf* FGC was also absent in the avian-linked serovars Javiana and Heidelberg. There were some serovar biases with pseudogene distribution for these core fimbriae, and pseudogenes were more likely to be present in the FGCs of host-restricted serovars. In contrast to the core FGCs, the *stf, lpf, ste, stc, stj* FGCs were only partially conserved (i.e. present in 40%–80% of the serovars) and revealed different serovar distributions (Figure S4). The *stf, lpf, stj* FGCs were absent in serovar Typhi and clade 4 *Salmonella*, and *stj* was also absent in some clade 1 members (Figure S3). Both *ste* and *stc* were absent in clade 4, and *ste* was also absent in clade 1a *Salmonella*, which is mainly represented by serovar Typhimurium. Finally, more than half of the FGCs (*peg, tcf, sef, stk, fae, sdg, sta, pef, sdf, peh, stg, sdh, mrk, sde, sdi, sdj, sdk, sdl, sdd, sba, sbb, sbc, sdc*) were only found in a few *Salmonella* (Figure S4). Notably, some newly identified FGCs were present in only one serovar. The *sba, sbb, sbc* FGCs were only found in *S. bongori*, *sdc* and *sdd* only in *S. enterica* subspecies *arizonae*, *sdi, sdj, sdk, sdl* only in subspecies *diarizonae*, *mrk* only in serovar Montevideo and *sde* only in serovar Tennessee. These results suggest that these FGCs were acquired more recently than the FGCs present in most *Salmonella*, as discussed later.

Using the currently available data, *Salmonella* have an average of 12 FGCs per strain. The collection of *Salmonella* fimbriae is represented by three groups of FGCs. A group of core FGCs that are shared by over 80% of the strains or serovars can be distinguished from a group of partially conserved FGCs that are shared by 40%∼80% of the serovars. A third group of FGCs consisted of sporadic FGCs that were shared by less than 40% of the serovars and included more than half of all the *Salmonella* FGCs.

### Classification of the FGCs in the *Salmonella* Fimbriome

As usher proteins are the most conserved FGC proteins, a phylogenetic tree was built by comparing the usher proteins from all the *Salmonella* FGCs. This tree, which was based on 950 proteins, was consistent with a tree built previously for 189 usher proteins that originated primarily from *Proteobacteria* [34]. This study confirmed that the *Salmonella* ushers were distributed in all the described clades of fimbriae (γ, κ, π, β, α, σ), missing only participants in the γ2 sub-clade (Figure 1). All of the prevalent FGCs, namely *bcf, fim, lpf, sth* and *sti* belonged to the γ1 sub-clade. The *sde* FGC is a new addition to the γ3 sub-clade previously described to include only *sef* and *saf* in *Salmonella*. Many new members of the γ4 clade were identified (*peh*, *sdj*, *peg*, *sdg*, *sdh*, *sba*, *stk*, *sdd*, *sdf*, *sdl*, *mrk*) that with the known *stc, sta, stb* FGCs make this clade the most diverse in *Salmonella*. Notably, the *mrk* FGC was only detectable in serovar Montevideo. The *mrk* designation was used because its gene cluster organization mimics the one of the *Klebsiella pneumonia mrk* FGC, which suggests that this FGC has moved by HGT. The fimbriae encoded by *K. pneumonia mrk* were characterized by their capacity to mediate mannose-resistant bacterial agglutination of tannic acid-treated erythrocytes [60].

σ clade fimbria includes the *sdc* FGC, which was found only in *S. arizonae*. While its usher protein showed low sequence similarity with others in *Salmonella*, the Blastp search the NCBI non-redundant database indicated that its closest relative was present in the genomes of a few other *Enterobacteriaceae*, such as *Citrobacter rodentium*, *Escherichia fergusonii* and *Enterobacter hormaechei*, suggesting that this FGC was acquired by HGT.

The κ clade consisted of three FGCs, one located on a plasmid, *pef*, and two new FGCs, *fae* and *sbc*, that share similarities with the plasmid-encoded K88 and K99 FGCs from *E. coli* [61]. The *pef* FGC was only found in serovar Typhimurium and a few other serovars such as Choleraesuis, Paratyphi C and I,4, [5],12:i:-. The *Salmonella fae* usher protein shares 85% identity with the *E. coli* orthologous usher and is found only on *S. bongori*. The *sbc* FGC shares both a similar FGC organization and an usher protein that is 43% identical to the *E. coli* K99 usher. The average pairwise differences between the κ-fimbrial ushers of *S. enterica* isolates were similar to those observed for the *E. coli* usher proteins. Thus the *E. coli* and *Salmonella* κ-fimbriae most likely share the same ancestor. The data also suggested that over time, *E. coli* and *Salmonella* have exchanged FGCs belonging to the κ-fimbrial clade by interspecies conjugative transfer of plasmids that afforded some selective advantage.

Six different types of *Salmonella* FGCs belonged to the π-fimbriae, named for the protoype *E. coli* P fimbriae [34], *stf, ste and std* being new ones. Gene cluster organizations were shared between *std, sdk, sbb* and *sdl*, and between *stf* and *ste*, albeit the latter had additional distal subunit genes. The *std* FGC, which was the predominant one in *Salmonella*, was reported to be involved in bacterial binding to murine cecal mucosa and intestinal persistence [51], [62]. The *ste* FGC, which was only prevalent in clades 1, 2 and 3 of *S. enterica* subsp. I (Figure S3), has been described to participate in *Salmonella* colonization of chicken intestines [52]. The role of the other π FGCs and their fimbriae remains unknown. The newly identified *sdk, sbb*, and *sdl* FGCs were only present in serovars typically isolated from cold-blooded animals (Figure S3, clade 5), suggesting that their function might be host specific.

The *stj* FGC was the only representative for the β-fimbriae, which was previously defined as fimbrial usher protein cluster 7 [63]. No structural or functional information is available for this small but distinct clade of FGCs, which lacks a typical adhesin gene, a characteristic assigned to fimbriae that assemble as thin fibrillae or nonfimbrial surface structures [34]. The α- fimbrial clade includes the alternative chaperone/usher FGC that was known as class 5 fimbriae [34]. The *tcf* is the only known α-fimbrial clade found in *Salmonella* and is present in several serovars.

Compared to the usher phylogenetic tree, the chaperone tree showed mostly similar lineages, particularly for the γ4, π and κclades (Figure S5). Some FGCs (e.g. *bcf* and *stb*) had two independent chaperone genes. One of the two *bcf* chaperone genes was located in another lineage, suggesting that the latter gene was once acquired by some recombination event. Curiously, the two *stb* chaperone genes remained together in another lineage, resulting most likely from both duplication and recombination events. Interestingly the *pehB* chaperone gene has two separate lineages for different serovars, suggesting divergent evolution in different serovar-specific environments or replacement by horizontal gene transfer.

In summary, the *Salmonella* pangenome carries a large diversity of fimbrial types, considering that it has fimbrial representatives of all six known fimbrial clades. Most core fimbriae belonged to the γclade, particularly the γ1 subclade, highlighting the adaptability and potential usefulness of this clade of FGCs for *Salmonella* survival and infection.

### Extensive Acquisition and Loss of FGCs during *Salmonella* Evolution

**Figure 2:**
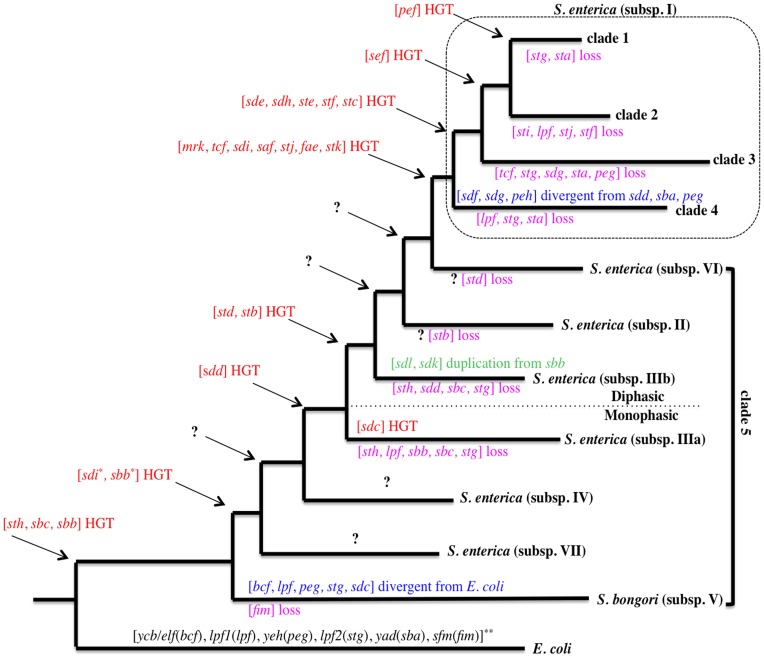
*Salmonella* and FGCs co-evolution model. Proposed tree that includes *E. coli* and the two *Salmonella* species, *S. bongori and* S. *enterica,* the latter being divided into seven subspecies (monophasic IIIa, IV, VII and diphasic I, VI, II, and IIIb; subsp. V is now *S. bongori*). FGCs shown in red are suggested to have been acquired by HGT. FGCs shown in blue have diverged from orthologous *E. coli* or other *Salmonella* FGCs. In purple are FGCs that were lost. In green are FGCs that were duplicated. A dotted line separates the subspecies based on the presence of one or two flagellin genes. A dotted frame includes all the *S. enterica* subsp. I. The 5 clades correspond to the ones shown in Figure S3. The asterisks indicate that *sdi* and *sbb* were found in the integrative and conjugative element ICESe3 region of *Salmonella enterica* subsp. VII strain SARC16, suggesting independent acquisitions of these FGCs.

A variety of studies have proposed phylogenetic trees to visualize the evolutionary history of *Salmonella* species and subspecies [24], [64], [65]. By integrating such data from previous microarray studies [66], [67] with our analysis of FGCs, we propose that an incremental set of steps can illustrate the acquisitions and losses of FGCs in *Salmonella* (Figure 2). When compared to *E. coli*, *Salmonella bongori* maintained five FGCs in the same genetic locus with few modifications by mutation and positive selection. The genes of these FGCs (*fim*, *stg*, *sba*, *peg*, *lpf*) were orthologs of the ones of the *E. coli* FGCs (*sfm*, *lpf2*, *yad*, *yeh*, *lpf1*), with *bcf* (*ycb*/*elf* in *E. coli*) having acquired an additional chaperone [34] (Table S1). A major event that resulted in the divergence of *Salmonella* from *E. coli* was the acquisition of SPI-1 [68]. This event enabled *Salmonella* to efficiently invade mammalian intestinal epithelial cells. The presence of new FGCs in *Salmonella* as compared to *E. coli*, suggests that the *sth*, *sbb*, *sbc* (Figure 2) clusters were also selected at this evolutionary step. Moreover, the similarity of the *sbc* FGC with the *fan* FGC, which is located on plasmids and encodes the K99 fimbriae of bovine or porcine enterotoxigenic *E. coli*, is suggestive of either an ancestral HGT gain accompanied of a loss during *S. enterica* evolution, or FGC acquisition after *Salmonella* speciation. Only *S. enterica* carries the *fim* FGC, as suggested by the currently available genomes and DNA hybrization data for *S. enterica* subsp. VII and IV [66]. Recent sequencing data (GenBank: FN298495.1) located the FGCs *sdi* and *sbb* in the integrative and conjugative element ICESe3 region of *S. enterica* subsp. VII, suggesting the two gene clusters were acquired by HGT (Table S2) [65]. Compared to *S. bongori*, *S. enterica* subsp. IIIa harbors two new FGCs, *sdc* and *sdd*, and lacks the *sth, lpf, sdb, sda, stg* FGCs indicating that these gains and losses occurred either at this step of evolution, or previously with subsp. VII or IV. *S. enterica* subsp. IIIb gained two new FGCs, *std* and *stb*. Whereas *std* was adjacent to a tRNA gene, *stb* had a sequence composition bias specific for pathogenicity islands, as determined by SIGI-HMM [69] (Table S2). As observed with subsp. IIIa, subsp. IIIb lost several FGCs. The *sth, sbc* and *stg* FGCs were lost by both subspecies, suggesting that they were lost before the speciation of IIIa and IIIb. The major evolutionary step that separates these two subspecies is the acquisition of a second flagellin locus by subsp. IIIb, allowing it to express either one of two flagellins thanks to a coordinated mechanism of flagellar phase variation between the two antigens (antigenic variation) [64], [66]. This more sophisticated system represents a selective advantage that likely occurred after the loss of these three FGCs.

Finally, the adaptive evolution of *Salmonella* from cold-blooded to warm-blooded animal hosts is characterized by the acquisition of SPI-2, which is only present in *S. enterica* subsp. I, as previously reported [15], [50]. Based on their acquisition and deletion of FGCs, we suggest an evolutionary pathway that has led to four clades of *S. enterica* subsp. I. First, seven FGCs were acquired (*mrk*, *tcf*, *sdi*, *saf*, *stj*, *fae*, *stk*), forming clade 4, which also lost three FGCs (*lpf*, *stg* and *sta*) and had three replaced, as determined by their analogous genomic loci (*sdd, sba* and *peg* for *sdf, sdg* and *peh*, respectively). As shown in Figure 2, clades 3, 2 and 1, were characterized by the gain of four or one, and the loss of five, four or two FGCs. Clades 3 and 4 include relatively rare serovars that were involved in recent food-borne disease outbreaks in the USA, such as Serovar Tennessee and Schwarzengrund in 2007, Agona in 2008 and Montevideo in 2009 to 2010 [70], [71](Figure S3). Clade 2 includes mainly the human-restricted Serovar Typhi and Parayphi A, and the serovar Newport strain SL254 which carries a multiple drug resistance plasmid [72]. This clade acquired only *sef*, while the *sti, lpf, stj* and *stf* FGCs were deleted. Clade 1 includes a greater diversity of serovars and FGCs, although serovar lineages tended to carry the same set of FGCs (Figure S3). This clade includes serovars Typhimurium and Enteriditis, the most common serovars responsible for human foodborne disease. A feature of the clade 1 serovars was the lack of *stg* and *sta* and for some them, the gain of *pef*.

The variable collections of FGCs in the different *Salmonella* species, subspecies and serovars resulted from complex changes that mainly involved FGC deletion and acquisition or replacement mediated by HGT, and to a lesser extent, duplication, and divergence. Additional genomic data, particularly for the *Salmonella* groups currently represented by only one genome, will help to improve evolutionary models in the future.

### *Salmonella* FGCs’ Evolution by Gene Duplication, Rearrangement and Divergence

A minimum of three genes is essential for the expression of a fimbria. These genes have to encode a periplasmic chaperone and an outer membrane usher protein required for fimbrial biogenesis, as well as a fimbrial subunit capable of assembling as a homopolymeric organelle on the bacterial surface [39]. Based on the phylogenetic tree of usher proteins, it is suggested that the different clades of *Salmonella* FGCs have evolved from such an ancestral FGC (Figure S6). Unlike other *Enterobacteriaceae*, none of the sequenced *Salmonella* harbors a minimal FGC with only three genes. The smallest *Salmonella* FGCs consist of four genes, the additional gene encoding a second fimbrial subunit with the characteristics of an adhesin. As shown with the *tcf* FGC of the α- clade (Figure S6), a subunit gene that has twice the size of other subunits typically encodes a minor fimbrial tip adhesin. Subunit duplication and recombination events might explain the relocation of the fimbrial adhesin gene at the 3′ end of the operon. Such a genetic organization is consistent with an evolutionary process for low-level expression of minor fimbrial subunits, including fimbrial tip adhesins that are predictably expressed in equimolar concentration with the usher. The *Salmonella* α-clade has only one representative resulting conceivably from gene rearrangement. In general, trans-complementation between genes of different FGCs in the same genome does not occur, due to the constraints of specific protein interactions during fimbrial biogenesis. Thus, the accumulation of new genes in FGCs is most likely the result of internal gene duplication and not of the acquisition of genes from other FGCs. The *tcf* FGC has an unusual gene organization with its chaperone at its 5′-end, suggesting a unique type of discriminatory gene regulation to ensure sufficient expression of the fimbrial subunit. Similarly, FGCs of the γ1, γ3 and β clades have evolved using gene duplication and sequence divergence. In addition the inversion of the subunit, chaperone and usher genes in the *sde* FGC found only in serovar Tennessee highlights an organizational trend that was maintained in the π and κ clades. The switched location of the fimbrial subunit gene at the end of the FGC suggests the presence of an additional promoter to ensure efficient subunit expression, as described for the *Salmonella sda*-like *Escherichia coli* K99 (*fan*) FGC [73]. Evolution of a basic fimbrial operon design into a more complex FGC with additional promoters might have benefited regulatory fine-tuning of fimbrial biogenesis. Such an evolutionary step is more likely the by-product of duplication, recombination and divergence than horizontal gene transfer, which would require structural adaptation of a foreign subunit to an evolutionary separate FGC. Whereas some π clade members maintained an ancestral type of FGC with only four genes, the *ste* and *stf* showed dramatic subunit duplication of distal subunits. The κ clade together with one branch of the γ4 highlight an evolutionary process of multiple subunit duplication and divergence steps at the 3′ end of FGCs. Interestingly, the other γ4 branch maintained a basic 4 gene operon-like organization, whereas one FGC, *stb*, gained an additional chaperone and subunit at the distal end of the FGC. The presence of such additions suggests further evolutionary specialization towards subunit-specific chaperones, as first described for the *E. coli* 987P fimbriae [74].

Although FGC-specific regulatory genes frequently flank a FGC, they can be found elsewhere on the genome. FGC-specific regulatory proteins are very diverse both in structure and mechanism of function, even for orthologous FGCs, indicating less stringent evolutionary linkage with the FGCs they regulate [75]. FGCs are frequently transcribed as one operational unit, an operon, with the first gene encoding the fimbrial subunit followed in sequence by the genes for the chaperone and usher proteins. One could speculate that FGCs have evolved out of an adhesive autotransporter protein [76], whereby gene fragmentation would have separated its three domains into an exported adhesive amino-terminal end (passenger domain) a central region (autochaperone domain) and a carboxy-terminal outer membrane channel (translocator domain). The genetic organization of FGCs permits basic regulatory mechanisms, such as rho-independent stem-loops located at the 3′-end of the subunit gene, to ensure that the structural subunit is expressed in larger amounts than the biogenesis proteins [75].

In summary, FGC comparisons present evidence of subunit gene duplication and gene order reorganization within FGCs as important mechanisms of FGC evolution. Subunit gene duplications can be associated with environmental adaptation and increased function fitness [77], [78]. The varied organization of FGCs is consistent with the selfish operon concept, whereby HGT of complete FGCs together with reorganization of operons increased the efficiency of gene co-regulation to benefit FGC survival [79].

### Functional Diversity Mediated by the Strain-specific Collection of FGCs

**Figure 3:**
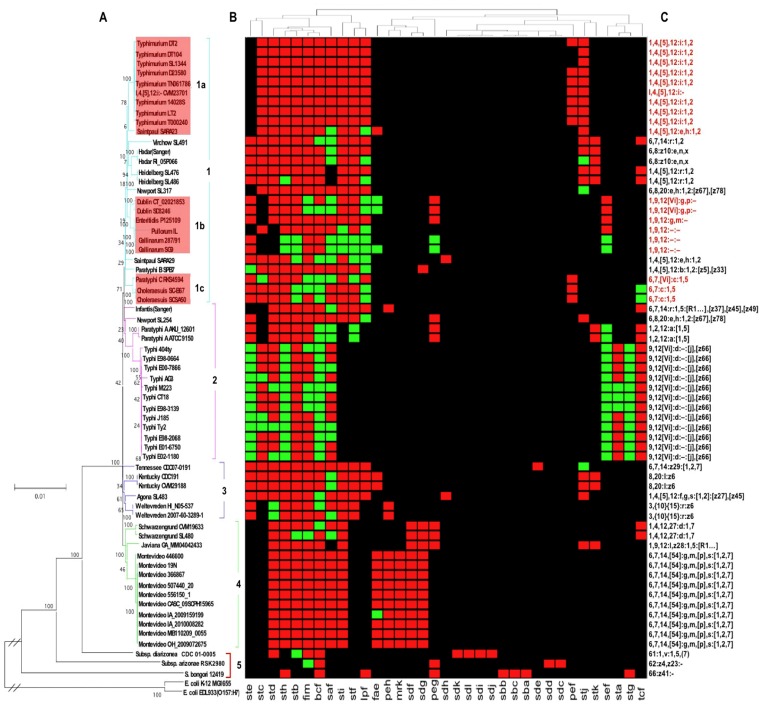
Correlation of *Salmonella* phylogenomic groups with specific collections of FGCs. On the left, phylogenomic tree of 90 *Salmonella* and two *E. coli* control strains, based on 45 highly conserved house-keeping genes totaling ∼43 Kb. Clade 1 to 4 correspond to the clades shown in Figure 2, and clade 5 includes the few sequenced genomes from strains that were not *S. enterica,* subsp. I. The scale indicates the number of substitutions per nucleotide. On the top and heat map, hierarchical clustering support tree for the FGCs (MeV, complete linkage method with an euclidean distance threshold of 9.525, http://www.tm4.org). FGCs with or without pseudogenes were shown as green or red rectangles, respectively. On the right, *Salmonella* serovars (somatic O and flagellar H antigens).

Over 99% of the human cases of salmonellosis are due to serovars that belong to the four clades of subsp. I. Clade 4 (Figure 3) includes serovar Montevideo, Schwarzengrund and Javiana that are commonly isolated in association with edible plants, such as red and black pepper, dehydrated chili, and tomato [70], [80]. This suggests that these serovars might have efficient mechanisms such as specific adhesive properties for long-term survival in the environment. Several clade 3 serovars are frequently isolated from edible products. For example, serovar Weltevreden strain 2007-60-3289-1 was isolated from a vegetable [81], [82], and serovar Tennessee was linked to a peanut butter outbreak in 2006–7 [83]. Curiously, serovar Tennessee has often been linked to urinary tract infections [84]. Several FGCs were specific for certain clade 4 serovars. For example, even though the *sdg* and *sdf* FGCs were typically present in all clade 4 serovars, the *mrk*, *peh*, *fae* were more specifically found in serovar Montevideo. The *K. pneumoniae* Mrk fimbriae has been described to bind to plant roots [85] as well as to extracellular matrix proteins and epithelial cells from the respiratory and urinary tracts. Serovar Montevideo and Kentucky carry the *fae* FGC, so designated for its similarity with the K88 FGC of enterotoxigenic *E. coli* [61] known to bind to several calf intestinal receptors [86]. With the exception of *mrk*, whether any of the other clade 3 and 4 specific FGCs encode plant-adhesive fimbriae that are expressed in an agricultural ecosystem is not known. Clade 2 of subsp. I. includes the human-specific serovar Typhi and Paratyphi A. Among serovar Typhi- and Paratyphi A-specific FGCs, including *tcf, sta, sef* and *stg*, only the *tcf* FGC was not degraded in some or all the strains. Genome degradation is a general outcome in both serovars and has been related to human host-restriction. The clade 1 includes both host-restricted and non-restricted serovars involved in human and animal gastroenteritis and septicemia. Most of these serovars share core FGCs (*saf, bcf, fim, stb, sth* and *std*), together with *sti, stf* and *lpf*, which are prominently absent in the clade 2 serovar Typhi.

Based on FGC sets, clade 1 (Figure 3) was subdivided in three subclades. Most of the broad host range serovar Typhimurium strains and strains of the two close relatives I,4, [5],12:i:- str. CVM23701 and serovar Saintpaul str. SARA23 shared essentially the same FGCs, with only *pef* lacking in a few strains, to constitute subclade 1a. This collection of FGCs might participate in the broad-host range of subclade 1a serovars. The *stc* and *lpf* FGCs were found to participate in long-term persistence in infected mice [87], which should promote *Salmonella* transmission. A separate branch of the clade 1 cluster that consisted of serovars Virchow, Hadar, and Heidelberg had the highest numbers of FGCs among all the serovars. These serovars carried the additional *ste, stk, fae* and *tcf* FGCs. Accumulation of a large number of different FGCs may benefit survival and transmission and broaden the host and environment range that can be colonized. It may also improve the efficiency of specific host colonization and result from active HGT and recombination events in such hosts.

Serovar Dublin, Enteritidis, Pullorum and Gallinarum, share similar O-antigens and FGCs, forming subclade 1b. As reported for many genes of host-restricted serovars, such as serovar Typhi [88], extensive gene degradation was also detected in the FGCs of the avian-restricted serovar Gallinarum, and to a lesser extent Pullorum (Figure 3). Although serovar Dublin is known to cause diarrhea, septicemia and abortion in cattle and serovar Enteritidis is a major avian colonizer, both serovars infect other animals including humans. These two serovars showed less gene degradation in their FGCs than the avian-restricted serovars and included the additional FGC *std* and *peg*. Only serovar Dublin and Enteritidis had a non-degraded *sef* FGC. The fimbriae of this FGC bind to murine intestinal epithelial cells and are involved in systemic murine and avian infections. Whether Sef also acts as a virulence factor in cattle remains to be determined. Since serovar Dublin and Enteritidis share the same FGCs, other factors might contribute to their respective preferential hosts. Comparisons of gene degradation in the FGCs of subclade 1c isolates indicated more similarities between the human isolates of serovar Choleraesuis and Paratyphi C [89], [90] than with the swine isolate of serovar Choleraesuis [91]. Even though all strains of Choleraesuis are thought to be capable of causing generalized disease in both human and swine, it would interesting to determine whether a subpopulation of serovar Choleraesuis, together with a specific panel of FGCs, are host specialists for swine. Thus, the same host specificity by different serovars might be the result of convergent evolution [90].

Although there are over 2600 *Salmonella* serovars based on the O- and H- antigens, few are known to be host species specialists (i.e. specific for individual animal host species). This property has historically been emphasized to support the concept of host-serovar specificity, despite a lack of experimental data to explain at the molecular level how individual O- or H-antigens would determine host species specificity. The fact that these antigens are always expressed in vitro presented the opportunity to use this trait for diagnostic classification and epidemiology surveys. However, the exclusive focus on these antigens to identify a link between host specificity and population diversity in *Salmonella* can be misleading. Many serovars are not host specialists, indicating that affinity for specific host species may involve alternative bacterial pathways and surface molecules other than O- and H-antigens. These other surface molecules might serve as better genotypic predictors of host specificity. Bacterial surface ligands that interact only with specific host receptors have been particularly well documented in *E. coli*, such as the K88 and 987P fimbrial adhesins that mediate bacterial adhesion and colonization of pig intestines. We propose that the affinity of *Salmonella* strains for certain host species and tissues might be best determined by a collection of surface proteins with host-, tissue- and cell specific binding properties, such as the fimbriae. The variability in the number, identity and organization of FGCs in each *Salmonella* strain correlated with evolutionary processes that result from differential adaptation to a large variety of niches for survival. The high numbers of pseudogenes in otherwise undeleted FGCs suggests either recent inactivation of unnecessary or deleterious functions (particularly in host-restricted serovars), or the accumulation of FGCs for potential future use upon reactivation in a new environment. Although *Salmonella* have been reported to adhere to or invade plants by using non-specific binding factors such as cellulose and curli [92]–[94], *Salmonella* is better adapted to the intestinal environment of animals, particularly warm-blooded animals, where *Salmonella* multiplication is poorly hindered. Although contaminated edible plants might benefit the transmission of *Salmonella*, mammalian and avian intestines are likely a better location for the development of efficient adaptive evolutionary activities. The relatively low number of FGCs found in *Salmonella* bongori and in serovars that are mainly associated with cold-blooded animals, as shown for clade 5 in Figure 3, is in agreement with this. Clade 5 *Salmonella* have nine out of 17 FGCs that are unique by being not found in other clades. These FGCs, *sba, sbb* and *sbc* for *Salmonella bongori*, *sdc* and *sdd* for *S. enterica* subspecies IIIa, and *sdl*, *sdk*, *sdj and sdi* for *S. enterica* subspecies IIIb might be expressed at lower temperatures and be specific for intestinal receptors of reptiles or other cold- blooded animals. As more clade 5 *Salmonella* are sequenced, it is likely that other unique FGCs will be identified. In contrast, the *bcf* FGC was conserved in all *Salmonella* clades, albeit in a degraded form for the human serovars Typhi and Paratyphi A. Even though *bcf* expression might be deleterious to the maintenance of the serovars Typhi and Paratyphi A in humans, experimental evidence has indicated that it contributes to the colonization of bovine Peyer’s patches (PPs) and participates in gastrointestinal and long-term systemic infection in mice without murine PPs colonization [51], [95]. Presumably, either Bcf or Bcf receptor expression varies, depending on the different mammalian hosts and tissues encountered by *Salmonella*. Most of the other fimbriae that are partially shared between *S. bongori* and *S. enterica* (such as *fim, stb, sth, std, lpf, peg*, and *stg*) were shown to bind to intestinal epithelial cells or to participate in the colonization of mammalian or avian intestines [51], [52], [96]. The *sta* FGC was unable to direct bacterial adhesion or invasion of the human intestinal cell line INT-407. However, it remains possible that Sta fimbriae recognize mammalian or avian intestinal cell receptors that are absent on INT407. Even though cold-blooded animals are a reservoir for *S. bongori,* and *S. enterica* subsp. *arizonae* and *diarizonae*, these organisms may still have ligands that can colonize mammals. Whether any of these fimbriae play a role in human colonization or infection remains unclear.

Taken together, the phylogenomic analysis of all the sequenced *Salmonella* strains was mostly consistent with serovar and FGC profiles. However, the observed discrepancies that highlighted macro-evolutionary processes such as HGT-mediated acquisition of FGCs and functional (and likely structural) loss of FGCs that lead to host species specificity was more interesting. The data presented support the hypothesis that specific fimbriae are involved in determining preferential niches or hosts for *Salmonella* survival or transmission.

### Functional Diversity Mediated by the Strain-specific Collection of Adhesin Alleles

In addition to the macro-evolutionary mechanisms of FGC gain and loss in *Salmonella*, the detection of allelic variants among the known or predicted functional molecules of fimbriae, namely their adhesins, attests to the presence of additional adaptive micro-evolutionary pathways. Accordingly, we suggest that in addition to the phenotype mediated by serovar- or strain-specific sets of FGCs, the allelic variants of fimbrial adhesins influence the preferential or specific colonization of certain host species and possibly the form and extent of the disease (carrier state, gastrointestinal or systemic symptoms). This assertion is supported by several studies on FimH, the type 1 fimbrial adhesin of *Salmonella*. The original definition for the type 1 fimbriae was based on the lectin-like affinity for mannose residues. Since mannose is a carbohydrate that frequently participates in the decoration of animal glycoproteins, including membrane glycoproteins, these fimbriae have been observed to bind to many cell types. A recently described FimH receptor is glycoprotein 2, which is expressed on the apical plasma membrane of M cells, where it serves as a bacterial transcytotic receptor [97]. As previously determined with small sets of *Salmonella* strains and serovars, the sequence of the type 1 fimbrial adhesin FimH demonstrates allelic variation. These FimH variants modulate the binding properties of the fimbriae, not only by changing the affinity for mannose, but also by substituting mannose for other receptors [59]. For example, the serine of serovar Enteritidis in place of phenylalanine of serovar Typhimurium at residue 96 of the mature protein altered the mannose-binding properties of FimH from a low to a high adhesive form [56], whereas two different strains of serovar Typhimurium with asparagine or tyrosine at position 136 presented different mammalian cell binding properties [59], that corresponded to mannosylated substrate binding [98]. Furthermore, a threonine to isoleucine substitution of residue 56 in the FimH of serovar Gallinarum and Pullorum could explain why this protein didn’t bind mannose [58]. This and other substitutions in the FimH residue(s) of serovar Gallinarum and Pullorum correlated with an improved FimH-mediated bacterial binding of these serovars to avian leucocytes [59]. Allelic variation of FimH has also recently been shown to influence the catch-bond adhesive properties of the *Salmonella* type 1 fimbriae [98].

This study compared the FimH sequences from the 90 available full genomes, 17 individual sequences in GenBank and the recently sequenced FimH from clinical isolates [99], [100] and from the Duguid et al. collection [101]. A total of 67 different FimH alleles carrying amino acid substitutions were identified in *S. enterica* subsp. I (Figure S7). Even though many residue substitutions were randomly distributed, others clearly identified hotspots. An average distance tree separated the FimH alleles into six groups (color-coded in Figure S7). Group one consisted exclusively of one allele found in the 10 Typhi strains that could be distinguished from all the other serovars by having unique FimH substitutions at positions 35, 36, 39, 137 and 195. Group two included FimH alleles characterized by substitutions at positions 49, 52, 67 and 295. Group three is less well-defined and included FimH alleles that had frequently substitutions at positions 10, 67, 115, 212 and/or 226. The fifth cysteine at position 104 of the sequenced serovar Abortusovis may be the result of a sequencing error, given that fimbrial subunits typically include even numbers of cysteine residues paired as cystines, consistent with the oxidized environment of a bacterial surface. Group four included FimH alleles characterized by substitutions at positions 104, 109 and/or 115. Unlike serovar Typhi, broad host range serovars such as Typhimurium and Enteritidis were distributed in both group three and four, and showed extensive allelic variability for FimH, highlighting phylogenetic incongruence for broad host range serovars and FimH. Group five consisted exclusively of the four serovar Paratyphi B FimH alleles that could be distinguished from the other serovars by unique substitutions at position 267. Group six consisted of a single strain of serovars Aluchua, which was the only allele with a substitution at position 288.

**Figure 4:**
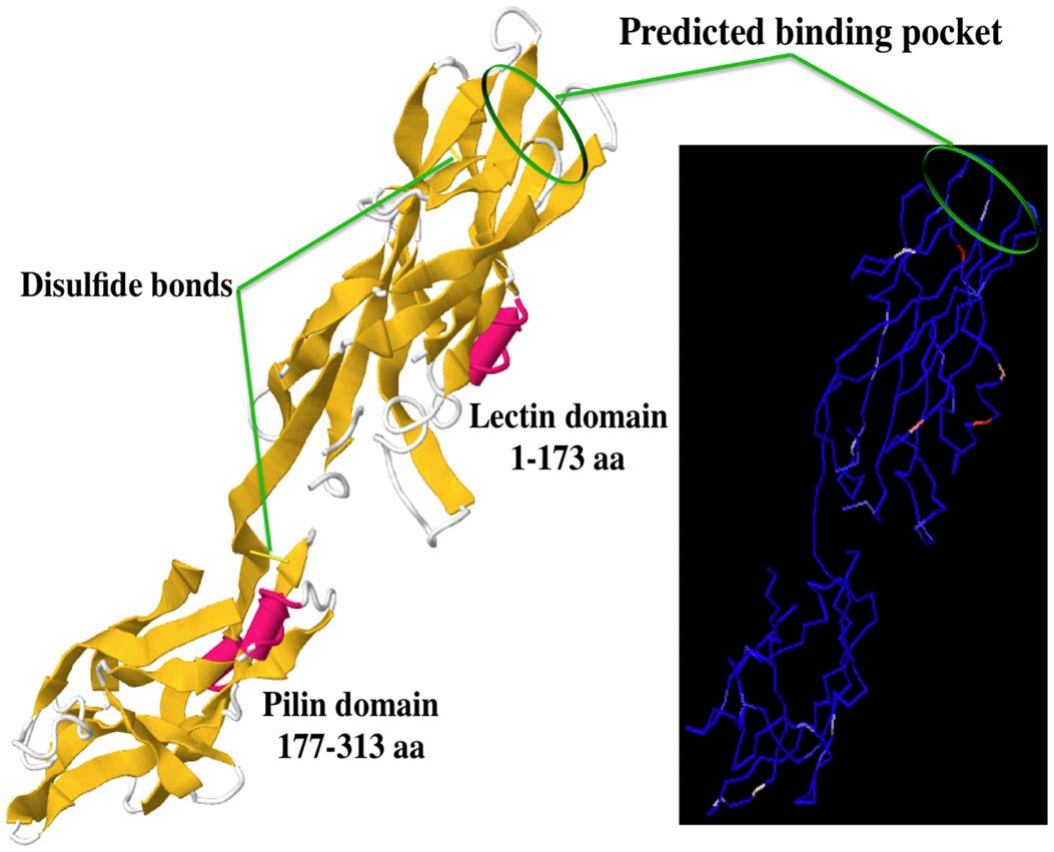
Predicted structural model of the *Salmonella* FimH fimbrial adhesin. The structure of the *Salmonella* FimH protein was based on the template structure 1klf (Protein Data Bank) from the *E. coli* FimH adhesin [119]. On the left, ribbon model of the predicted structure of *Salmonella* FimH with its lectin and pilin domains, each with one disulfide bond. ~β -barrel are shown in yellow and α-helices are shown in pink. On the right, the variable amino acid positions are shown in a tube-rendering model of the FimH backbone structure, with a color gradation from blue (most conserved residues) to red (most variable positions). None of the natural variable positions were located in the predicted binding pocket, shown as green circles on both models.

A three-dimensional model for the mature *Salmonella* FimH adhesin is proposed, based on the structure of the *E. coli* FimH protein (Figure 4). Both the amino-terminal residues 1 to 173 predicted to carry the binding pocket and the carboxy-terminal half predicted to function as the fimbrial assembly domain (residue 177 to 313) had similar numbers of substituted positions (29 versus 25, respectively). The linker region had two positions with substitutions. Even though most of these substitutions did not include the residues predicted by Phyre2 to interact with mannose in the binding pocket, residues 52, 56 and 155 were located in the loops that form the pocket (Figure 4). Only a few FimH with substitutions in a total of 56 variable positions from 67 natural alleles (Figure S7) were studied for their effects on adhesion. These FimH alleles had substitutions in 2, 3 or 5 different positions and their adhesiveness was increased, decreased or unaffected respectively.

Findings with natural FimH alleles were in agreement with those of a recent study that engineered a library of random *fimH* mutants by PCR [98]. In this study 15 out of 38 single mutants bound 2 to 7 times better than the parental strain to mannose-BSA, 10 of these mutants being dispersed along the amino-terminal half of FimH, with only one apparent hotspot at positions 136–138. Only two mutations (N136D and R232W) targeted residues that vary naturally (N136Y in Typhimurium strain AJB3, and R232W in Paratyphi C strain RK54954). Although most of the substitutions were in the lectin domain of FimH, only one was in a loop for the binding pocket (Y15F) and none included residues predicted to participate in the mannose-specific binding pocket itself. Since enhanced binding was mainly observed with substitutions located further away from the mannose-binding pocket, and more proximal to the predicted interface between the lectin and pilin domains of FimH, the authors suggested that allosteric effects were the prevalent modifiers of binding affinities [98]. Consistent with the *E. coli* FimH catch bond adhesin model [102], the binding affinity of a *Salmonella* FimH was increased by extending the binding molecule through shear force. Studies on a few other fimbriae supported this model [103], [104], suggesting that the evolution of fimbrial adhesins by allelic variation has benefited bacteria not only by increasing the range of receptor and host specificities, but also by modulating binding strengths to better resist environmental or host clearing mechanisms. Antibodies to the *E. coli* FimH lectin domain mainly recognize the extended high-affinity conformer of the protein and accordingly enhance binding of the fimbriae [105]. Consistent data were obtained with *Salmonella* [98], suggesting that new approaches will be needed to induce anti-adhesive antibodies, such as antibodies that bind preferentially to low-affinity conformers of adhesins known to function by a catch-bond mechanism of binding. The linear epitopes of FimH predicted to be most immunogenic include residues 49–56 and 115–155 [106], and thus include the binding pocket residues of FimH. The high-affinity conformers, by being more extended, may present more efficiently linear epitopes than the more compact low-affinity conformer, which may be better recognized by conformation-specific antibodies.

In contrast to a large body of studies on FimH, no other *Salmonella* fimbrial adhesin subunit has been identified and characterized functionally. Nevertheless, several fimbrial structures of *Salmonella* have been shown to provide adhesive properties with corresponding FGCs carrying one (or 2 for *ste* FGCs) predicted adhesin gene (Table S3). In addition to the *fim* FGC, the other *Salmonella* core FGCs showed a range of 19 to 25 allelic variants of their predicted adhesins for a total of 70 to 80 sequences (Table S4, in red). For the partially conserved FGCs (*stc, ste, stf, lpf, stj*) (Table S4, in green), 6 to 22 different alleles were found in the 18–36 available sequences, with SteG and LpfD showing the most variation at the protein sequence level. Most of the remaining FGCs had few detectable adhesin alleles, due to insufficient numbers of available sequences or serovars (Table S4, sporadic adhesins, in blue). Nevertheless, some of these adhesin alleles (SdbD, FaeG, TcfD, StgD) showed tremendous sequence variations between themselves, indicating that orthologous chaperone-usher genes share synteny with genes that encode highly variable subunits predicted to be adhesins. Interestingly, one FGC (*ste)* frequently carried two predicted adhesin genes in tandem, suggestive of gene duplication. In several strains, *ste* carried only one predicted adhesin gene with a size and composition that suggested the result of a recombination event between the two genes.

Taken together, studies on allelic variation of the FimH *Salmonella* adhesin have began to unravel how structure variability intervenes in the function of this ligand. Despite a great number of substituted amino acids in FimH, the restricted number of different alleles is likely representative of adaptive mutation and recombination events guided by beneficial functions of FimH for *Salmonella* survival in specific or diverse environments. Phylogenetic incongruence between serovars and FimH adhesins is consistent with evolutionary pathways that have been impacted by HGT and recombination. Allelic variation of other fimbrial ligands in *Salmonella*, as determined here with predicted fimbrial adhesins, should guide new studies aimed at determining the role of ligand diversity for the lifestyle and pathogenesis of *Salmonella*.

### Evolution of a Large Fimbriome and Adhesinome

The comparative analysis of *Salmonella* fimbrial adhesins undertaken in this study suggests that macroevolutionary pathways led to the gain of new adhesins by FGC duplication or/and HGT. Microevolutionary adaptation processes directed both the diversification of adhesin specificity and affinity by substitution mutagenesis, and the inactivation of genes that were incompatible with the new lifestyle of the strain [107]. Gene disruption was mostly detected in host-restricted serovars by the large number of fimbrial pseudogenes, as a result of frameshifts. Consistent with the interpretation of our data, lineage-specific positive selection of genes, including adaptive gene loss, has been documented to contribute to the evolution of host restricted *Salmonella* serovars [53], [88], [90], [108]. This study offered a glimpse into the genetic diversity of the *Salmonella* fimbriome and adhesinome (the collection of adhesins and adhesin alleles in the *Salmonella* pangenome) and evaluated the phylogeny of a large number of new *Salmonella* fimbriae (one third) that had not been detected and catalogued previously [34], [39]. More sequence data from *Salmonella* strains will be needed, particularly from underrepresented serovars, to explore the relationship between *Salmonella* adhesin type or allele and host or niche specialization. The current analysis should also instigate new in vitro and in vivo studies to improve our understanding of the role of most *Salmonella* fimbriae on this bacterium’s binding properties, lifestyle and choice of hosts. Intestinal adhesion mechanisms together with other virulence factors profit efficient *Salmonella* residence, multiplication and transmission to new hosts [32], [52], [62]. The detection of associations of collections of strain-specific fimbriae and adhesin alleles with host species and potential disease progression should support the development of new rational diagnostic and therapeutic approaches.

## Methods

### Data Collection and Annotation for *Salmonella* DNA

The available *Salmonella* chromosomal and plasmid genome sequences were obtained from NCBI (http://www.ncbi.nlm.nih.gov/bioproject/12302), the Welcome Trust Sanger Institute (http://www.sanger.ac.uk/resources/downloads/bacteria/salmonella.html), the Genome Institute at Washington University (http://genome.wustl.edu/genomes/P180/), and Salmonella.org (http://www.salmonella.org/genomics/). The data were from 90 chromosomal and 60 plasmid sequences from 115 *Salmonella* strains (Table S5). All the sequences not found in NCBI (Genbank) were submitted to the RAST annotation server (http://rast.nmpdr.org/) for standard genome annotation and targeted sequence extraction [109]. The latter sequences were from *S. bongori* 12149 (ATCC 43975) and *S. enterica* serovars Hadar, Infantis, Typhimurium DT104 and DT2 from the Welcome Trust Sanger Institute, serovar Pullorum from Salmonella.org, and *S. enterica* subspecies *diarizonae* CDC 01–0005, from the Genome Institute at Washington University. The list includes 114 *S. enterica* strains and only one *S. bongori* strain. 112 of the 114 *S. enterica* belong to the subspecies *enterica* (I), one to the subsp. *arizonae* (IIIa) and one to the subsp. *diarizonae* (IIIb). The list carries a total of 27 different serovars. From the 115 strains, only 90 have chromosomal sequences, 19 of them with 30 identified plasmids (1–3 plasmids per strain). The remaining 30-plasmid sequences originate from strains with unknown chromosomal sequences. In addition, the genomes of two *Escherichia coli* strains, *E. coli* K-12 substrain MG1655 and enterohemorrhagic *E. coli* O157:H7 EDL933 with accession number NC_000913.2 and NC_002655.2, respectively, were collected from NCBI to be used as control genomes to assemble a *Salmonella* phylogenomic tree.

### Identification of *Salmonella* Fimbrial Usher and Chaperone Proteins

Fimbrial gene clusters always encode a single usher protein, which is the largest and most conserved protein of a fimbrial apparatus. Thus, sequences of usher proteins were used to identify and collect all predicted genes and encoded proteins of fimbrial gene clusters from the available *Salmonella* genomes. At least one usher representative of each fimbrial gene cluster from the Virulence Factor Database (VFDB) (http://www.mgc.ac.cn/cgi-bin/VFs/genus.cgi?Genus=Salmonella) was used to search all the *Salmonella* genomes with BLASTp (http://www.ncbi.nlm.nih.gov/blast/Blast.cgi?) [110]. A similar search was repeated with chaperone proteins, which are the next most conserved proteins of fimbrial systems. Ushers and chaperones were recognized by alignment coverage of 70% with an *E*-value below 0.1. Hits that showed shorter alignments were checked manually to detect potential frameshifts and resulting pseudogenes in the context of fimbrial gene clusters. By using the same approach, we identified also the FGCs in two control *E. coli* strains to detect orthologs (Table S2).

### Characterization of Fimbrial Gene Clusters and Predicted Proteins

Usher and chaperone proteins, as well as proteins encoded by genes neighboring usher and chaperone genes (whether or not annotated as being part of a fimbrial system) were queried with the Conserved Domain Database v2.29 (http://www.ncbi.nlm.nih.gov/Structure/cdd/wrpsb.cgi) and the InterPro release v18.0 database (http://www.ebi.ac.uk/Tools/pfa/iprscan/). In addition, fimbrial proteins from newly identified fimbrial gene clusters were characterized by undertaking BLASTp searches with the non-redundant protein database of Genbank. Genes from clusters that shared synteny with fimbrial clustered genes in other *Enterobacteriaceae* were given the same designation, whereas genes from previously unnamed fimbrial gene clusters were given new designations (*sba, sbb*, *sbc*, *sdc*, *sdd, sde, sdf, sdg, sdh, sdi, sdj, sdk, sdl, peh*). To identify putative adhesins within *Salmonella* FGCs, we utilized unique characteristics of known adhesin genes in *Escherichia coli*. First, fimbrial subunit genes were recognized as genes flanking the usher or chaperone(s) genes and harboring specific sequence signature recognized by the sequence search tools used above. Second, the adhesin gene is typically larger than other subunit genes to accommodate a binding domain. Genes that encode fimbrial adhesins are frequently twice the size of structural (or pilin) subunit genes. This has been best exemplified with the resolved structure of tip adhesins, as well as with an internal adhesin [36], [111]–[113]. Third, adhesin genes typically follow the usher and chaperone genes in the directional order of transcription of an FGC and are frequently the most distal fimbrial subunit gene. Each identified FGC of *Salmonella* had at least two fimbrial subunit genes and most FGCs carried only one clearly predictable adhesin, with the exception of 1 FGC (*ste*), which had 2 ORFS that could encode a potential adhesin. Table S6 lists the genes and pseudogenes (labeled with asterisks) of all the FGCs, as well as their locus numbers.

### Evaluation of the Number of different FGCs in *Salmonella*

A mathematical model was used to evaluate the number of different FGCs in the pangenome of *S. enterica* subsp I. For this, only FGCs found in serovars that had strains with different sets of FGCs were used, namely, the 26 FGCs of serovars Dublin, Gallinarum, Newport, Saintpaul and Typhimurium. A constructed collector’s curve was found to near an asymptote. The curve was fitted to a logarithmic curve with the equation y = 4.2043 ln(x) +11.655 (r^2^ = 0.99957). The current number of identified *S. enterica* subsp I serovars being 1531 (x), a total number of different FGCs for this subspecies was predicted to be 42.5 (y).

### Phylogenetic Analysis of Fimbrial Usher and Chaperone Proteins

Protein sequences were chosen for phylogenic analysis because they are significantly more conserved than DNA sequences. Trees with DNA sequences include too much noise due to synonymous mutations. From a functional point of view, evolution of the protein sequences is more relevant, considering that fimbrial biogenesis involves many fimbriae-specific protein-protein interactions that restrict the selection of evolutionary mutations. Earlier phylogenic studies of bacterial fimbriae used the same approach [39], [40]. MEGA 5.05 was used to prepare multiple sequence alignments of 950 usher and 1094 chaperone proteins with ClustalW (default parameters) and to construct phylogenetic trees (Neighbor-Joining method with bootstrap replication 1,000, Jones-Taylor-Thornton model).

### Phylogenomic Analysis of *Salmonella*

A phylogenomic tree was prepared from the available 90 *Salmonella* genomes, along with the two *E. coli* reference genomes described above. To identify the orthologous genes shared by these bacteria, their predicted encoded protein sequences were retrieved from the GenBank and RAST databases. Pairs of proteins with more than 45% identity, 70% alignment coverage, and an E-value below 1×10^−20^ were considered orthologous. To construct the phylogenomic tree, 45 highly conserved orthologous genes involved in gene regulation and transcription were chosen (*cheB*, *cheR*, *clpS*, *cobB*, *comEA*, *fis*, *flgM*, *flhD*, *ftsB*, *ftsY*, *groS*, *grxB*, *helD*, *holC*, *hscB*, *hslR*, *infB*, *luxS*, *metJ*, *mreC*, *ndaG*, *nikR*, *nrdR*, *nusA*, *prmA*, *rho*, *rib*, *rimM*, *rnhA*, *rof*, *rplQ*, *rplY*, *rpoN*, *rpsP*, *rsd*, *rsmE*, *sulA*, *tsf*, *ybeA*, *yciH*, *yfjA*, *yhbY*, *yhhF*, *yhjY*, and *yicC*). For this, the 45 genes were concatenated into a nucleotide sequence of approximately 43 kb. The 92 concatenated sequences were aligned using ClustalW with default parameters to produce an alignment in MEGA format [114]. Phylogenomic Tree construction was done using the Maximum-likelihood method with a bootstrap value of 1,000 [27]. To evaluate the distribution of fimbrial gene clusters among the *Salmonella* spp., the MultiExperiment Viewer was used to make a hierarchical clustering support tree (MeV4.7, http://www.tm4.org).

### FimH Allele Analysis and Structural Modeling

In addition to the FimH sequences from the 90 available *Salmonella* genomes, *Salmonella* FimH sequences were collected from GenBank and recent publications [99] [100], [101]. FimH allele groups were characterized by the average distance tree that was produced with BLOSUM62 (http://www.ebi.ac.uk/Tools/msa/clustalw2/). Alignments of the alleles were obtained with ClustalW and further edited manually. Structure modeling of the FimH sequences was done with Phyre-2 (http://www.sbg.bio.ic.ac.uk/phyre2/html/page.cgi?id=index) [115], nFOLD3 (http://www.reading.ac.uk/bioinf/nFOLD/) [116], MUSTER (http://zhanglab.ccmb.med.umich.edu/MUSTER/) [117] and I-TASSER (http://zhanglab.ccmb.med.umich.edu/I-TASSER/) [118], respectively. The best structural template (1klf, Protein Data Bank, *E. coli* FimH) [119] was used to construct the tertiary structure of FimH using Phyre-2. The predicted model was viewed and analyzed using the molecular visualization program Jmol (http://jmol.sourceforge.net/). The protein variability server was used to analyze FimH allele variability within a multiple of sequence alignment and mapping on the structure [120]. Residues involved in the putative binding site of the FimH lectin domain were discerned by using the eF Site database [121] and searching for large cleft with the Pocket program of Phyre-2. A FimH sequence was sent to the IEDB analysis resource for identification of epitope sequences and 3D structural homology mapping [106]. Other adhesion and putative adhesin sequences (Table S7) were sent to the Datamonkey server, using the TOGGLE model to determine the number of different alleles with substitution sites and types [122].

## Supporting Information

Figure S1 Relative numbers of FGCs per strain or per serovar. The horizontal axis shows the numbers of fimbriae per strain, and the vertical axis shows the corresponding percentages of strains (blue) or serovars (red) for each number.
(TIF)

Figure S2 Collector’s curve for the prediction of the total number of different FGCs in *S. enterica* subsp. I. The 26 FGCs of serovars Dublin, Gallinarum, Newport, Saintpaul and Typhimurium were used to construct the curve. The obtained curve was fitted to a logarithmic curve with the equation y = 4.2043 ln(×) +11.655 (r^2^ = 0.99957).
(TIF)

Figure S3 Evolution of *Salmonella* along with their FGCs. On the left, phylogenomic tree of 90 *Salmonella* derived from 45 highly conserved house-keeping genes (see Methods) totaling ∼43 Kb. All the *Salmonella* formed five distinct clades labeled 1 to 5 (as shown in Figures 2 and 3). The scale indicates the number of substitutions per nucleotide. Heat map, distribution of FGCs; FGCs with or without pseudogenes were shown as green or red rectangles, respectively. On the top, phylogenetic tree for the FGCs, adopted from Figure 1.
(TIF)

Figure S4 Relative frequency distribution for each FGC. The horizontal axis shows each of the 35 distinct FGCs and the vertical axis shows corresponding percentages of strains (blue) or serovars (red) for each FGCs. The data were from 90 strains covering 27 serovars.
(TIF)

Figure S5 Phylogenic tree for *Salmonella* fimbrial chaperones. A phylogenetic tree was built for all identified FGCs in the *Salmonella* pangenome by using the amino acid sequences of the combined 1094 chaperone proteins (MEGA 5.0, as described in method). The FGCs were divided into five clades and the genes were color-coded as shown in Figure 1. The scale indicates the number of substitutions per amino acid.
(TIF)

Figure S6 Evolution model for the *Salmonella* fimbriome. The proposed evolution pathway (and color code) is based on the FGC classification shown in Figure 1 and starts at the bottom of the figure with a prototypical ancestral FGC.
(TIF)

Figure S7 FimH alleles in *Salmonella* subspecies I. Available genomic and protein sequence data identified 67 FimH alleles in *Salmonella* subspecies I. The alleles were divided in six groups (1 to 6, on the left) based on a FimH average distance tree produced by using BLOSUM62. The top two rows list the substituted FimH residue positions with the most prevalent residue at this position. The background for the lectin domain residues is labeled in dark gray, whereas the background of the pili domain residues is labeled in light gray (linker domain in white). Residue positions in red correspond to mannose-binding enhancing substitutions and residue positions in green correspond to mannose-binding neutral substitutions; residue substitution effects on mannose binding in other positions is not known.
(TIF)

Table S1 FGCs of two strains of *E. coli* and their orthologous FGCs in *Salmonella*.
(XLS)

Table S2 *Salmonella* FGCs and characteristics supportive of past HGT for some FGCs.
(XLS)

Table S3 Genes of each FGC and known functional phenotypes. Known or predicted adhesin genes are shown in red.
(XLS)

Table S4 Variation of *Salmonella* fimbrial core (red), conserved (green) and sporadic (blue) adhesin genes (or predicted adhesin genes).
(XLS)

Table S5 List of *Salmonella* strains that provided genomic or plasmid sequence information.
(XLS)

Table S6 Gene loci for all the genes in all the *Salmonella* FGCs of the genomic data.
(XLS)

Table S7 GenBank accession numbers for *Salmonella* fimbrial adhesin genes that were not from genomic data.
(XLS)

## References

[1] Hirsh DC, Hirsh DC, MacLachlan NJ, Walker RL (2004). *Enterobacteriaceae: Salmonella*..

[2] Crump JA, Mintz ED (2010). Global trends in typhoid and paratyphoid Fever.. Clin Infect Dis.

[3] Pang T, Bhutta ZA, Finlay BB, Altwegg M (1995). Typhoid fever and other salmonellosis: a continuing challenge.. Trends Microbiol.

[4] CDC (2009). Preliminary FoodNet Data on the incidence of infection with pathogens transmitted commonly through food–10 States, 2008.. MMWR Morb Mortal Wkly Rep.

[5] Scallan E, Hoekstra RM, Angulo FJ, Tauxe RV, Widdowson MA (2011). Foodborne illness acquired in the United States-major pathogens.. Emerg Infect Dis.

[6] CDC (2011). Surveillance for foodborne disease outbreaks – United States, 2008.. MMWR Morbidity and mortality weekly report.

[7] Dallap Schaer BL, Aceto H, Rankin SC (2010). Outbreak of salmonellosis caused by *Salmonella enterica* serovar Newport MDR-AmpC in a large animal veterinary teaching hospital.. J Vet Intern Med.

[8] Cummings KJ, Divers TJ, McDonough PL, Switt AM, Wiedmann M (2010). Temporal clusters of bovine *Salmonella* cases at a veterinary medical teaching hospital, 1996–2007.. Vector Borne Zoonotic Dis.

[9] Mateus A, Taylor DJ, Brown D, Mellor DJ, Bexiga R (2008). Looking for the unusual suspects: a *Salmonella* Dublin outbreak investigation.. Public Health.

[10] Muhammad M, Muhammad LU, Ambali AG, Mani AU, Azard S (2010). Prevalence of *Salmonella* associated with chick mortality at hatching and their susceptibility to antimicrobial agents.. Vet Microbiol.

[11] Anderson LA, Miller DA, Trampel DW (2006). Epidemiological investigation, cleanup, and eradication of pullorum disease in adult chickens and ducks in two small-farm flocks.. Avian Dis.

[12] Huang TM, Lin TL, Wu CC (2009). Serovar distribution and antimicrobial susceptibility of swine *Salmonella* isolates from clinically ill pigs in diagnostic submissions from Indiana in the United States.. Lett Appl Microbiol.

[13] You Y, Rankin SC, Aceto HW, Benson CE, Toth JD (2006). Survival of *Salmonella enterica* serovar Newport in manure and manure-amended soils.. Appl Environ Microbiol.

[14] McDonough PL (2009). Report of the Committee on *Salmonella*. Town and Country Resort and Convention Center San Diego, California.. United States Animal Health Association.

[15] Baumler AJ (1997). The record of horizontal gene transfer in *Salmonella*.. Trends in microbiology.

[16] Tindall BJ, Grimont PA, Garrity GM, Euzeby JP (2005). Nomenclature and taxonomy of the genus *Salmonella*.. International journal of systematic and evolutionary microbiology.

[17] Grimont PAD, Weill F-X (2007). Antigenic Formulae of the *Salmonella* Serovars.. WHO Collaborating Center for Reference and Research on Salmonella.

[18] Reeves P (1993). Evolution of *Salmonella* O antigen variation by interspecific gene transfer on a large scale.. Trends in genetics : TIG.

[19] Li J, Nelson K, McWhorter AC, Whittam TS, Selander RK (1994). Recombinational basis of serovar diversity in *Salmonella enterica*.. Proceedings of the National Academy of Sciences of the United States of America.

[20] Wildschutte H, Wolfe DM, Tamewitz A, Lawrence JG (2004). Protozoan predation, diversifying selection, and the evolution of antigenic diversity in *Salmonella*.. Proceedings of the National Academy of Sciences of the United States of America.

[21] Atterbury RJ, Van Bergen MA, Ortiz F, Lovell MA, Harris JA (2007). Bacteriophage therapy to reduce *Salmonella* colonization of broiler chickens.. Applied and environmental microbiology.

[22] Baumler AJ, Hargis BM, Tsolis RM (2000). Tracing the origins of *Salmonella* outbreaks.. Science.

[23] Pabbaraju K, Miller WL, Sanderson KE (2000). Distribution of intervening sequences in the genes for 23S rRNA and rRNA fragmentation among strains of the *Salmonella* reference collection B (SARB) and SARC sets.. Journal of bacteriology.

[24] McQuiston JR, Herrera-Leon S, Wertheim BC, Doyle J, Fields PI (2008). Molecular phylogeny of the salmonellae: relationships among *Salmonella* species and subspecies determined from four housekeeping genes and evidence of lateral gene transfer events.. Journal of bacteriology.

[25] Lan R, Reeves PR, Octavia S (2009). Population structure, origins and evolution of major *Salmonella enterica* clones.. Infection, genetics and evolution : journal of molecular epidemiology and evolutionary genetics in infectious diseases.

[26] Litrup E, Torpdahl M, Malorny B, Huehn S, Christensen H (2010). Association between phylogeny, virulence potential and serovars of *Salmonella enterica*.. Infection, genetics and evolution : journal of molecular epidemiology and evolutionary genetics in infectious diseases.

[27] Delsuc F, Brinkmann H, Philippe H (2005). Phylogenomics and the reconstruction of the tree of life.. Nature reviews Genetics.

[28] Stevens MP, Humphrey TJ, Maskell DJ (2009). Molecular insights into farm animal and zoonotic *Salmonella* infections.. Philos Trans R Soc Lond B Biol Sci.

[29] Rabsch W, Andrews HL, Kingsley RA, Prager R, Tschape H (2002). *Salmonella enterica* serotype Typhimurium and its host-adapted variants.. Infect Immun.

[30] Schlumberger MC, Hardt WD (2006). *Salmonella* type III secretion effectors: pulling the host cell’s strings.. Curr Opin Microbiol.

[31] Korhonen TK, Rhen M, Maskell D, Mastroeni P, Threlfall J (2007). Adhesins of *Salmonella* and their putative roles in infection..

[32] Humphries AD, Townsend SM, Kingsley RA, Nicholson TL, Tsolis RM (2001). Role of fimbriae as antigens and intestinal colonization factors of *Salmonella* serovars.. FEMS Microbiol Lett.

[33] van der Velden AW, Baumler AJ, Tsolis RM, Heffron F (1998). Multiple fimbrial adhesins are required for full virulence of *Salmonella typhimurium* in mice.. Infect Immun.

[34] Nuccio SP, Baumler AJ (2007). Evolution of the chaperone/usher assembly pathway: fimbrial classification goes Greek.. Microbiol Mol Biol Rev.

[35] Waksman G, Hultgren SJ (2009). Structural biology of the chaperone-usher pathway of pilus biogenesis.. Nat Rev Microbiol.

[36] Van Molle I, Joensuu JJ, Buts L, Panjikar S, Kotiaho M (2007). Chloroplasts Assemble the Major Subunit FaeG of *Escherichia coli* F4 (K88) Fimbriae to Strand-swapped Dimers.. J Mol Biol.

[37] Van Loy CP, Sokurenko EV, Moseley SL (2002). The major structural subunits of Dr and F1845 fimbriae are adhesins.. Infection and immunity.

[38] Khan AS, Johnston NH, Goldfine H, Schifferli DM (1996). Porcine 987P glycolipid receptors on intestinal brush borders and their cognate bacterial ligands.. Infect Immun.

[39] Zav’yalov V, Zavialov A, Zav’yalova G, Korpela T (2010). Adhesive organelles of Gram-negative pathogens assembled with the classical chaperone/usher machinery: structure and function from a clinical standpoint.. FEMS Microbiol Rev.

[40] Nuccio S-P, Thomson N, Fookes M, Bäumler AJ, Porwollik S (2010). Fimbrial signature arrangements in *Salmonella*..

[41] La Ragione RM, Coles KE, Jorgensen F, Humphrey TJ, Woodward MJ (2001). Virulence in the chick model and stress tolerance of *Salmonella enterica* serovar Orion var. 15+.. Int J Med Microbiol.

[42] Barnhart MM, Chapman MR (2006). Curli biogenesis and function.. Annu Rev Microbiol.

[43] Saldana Z, Xicohtencatl-Cortes J, Avelino F, Phillips AD, Kaper JB (2009). Synergistic role of curli and cellulose in cell adherence and biofilm formation of attaching and effacing *Escherichia coli* and identification of Fis as a negative regulator of curli.. Environ Microbiol.

[44] Kingsley RA, Humphries AD, Weening EH, De Zoete MR, Winter S (2003). Molecular and phenotypic analysis of the CS54 island of *Salmonella enterica* serotype Typhimurium: identification of intestinal colonization and persistence determinants.. Infect Immun.

[45] Kingsley RA, Santos RL, Keestra AM, Adams LG, Baumler AJ (2002). *Salmonella enterica* serotype Typhimurium ShdA is an outer membrane fibronectin-binding protein that is expressed in the intestine.. Mol Microbiol.

[46] Kingsley RA, van Amsterdam K, Kramer N, Baumler AJ (2000). The *shdA* gene is restricted to serotypes of *Salmonella enterica* subspecies I and contributes to efficient and prolonged fecal shedding.. Infect Immun.

[47] Dorsey CW, Laarakker MC, Humphries AD, Weening EH, Baumler AJ (2005). *Salmonella enterica* serotype Typhimurium MisL is an intestinal colonization factor that binds fibronectin.. Mol Microbiol.

[48] Jose J, Meyer TF (2007). The autodisplay story, from discovery to biotechnical and biomedical applications.. Microbiol Mol Biol Rev.

[49] Townsend SM, Kramer NE, Edwards R, Baker S, Hamlin N (2001). *Salmonella enterica* serovar Typhi possesses a unique repertoire of fimbrial gene sequences.. Infect Immun.

[50] Edwards RA, Olsen GJ, Maloy SR (2002). Comparative genomics of closely related *Salmonellae*.. Trends Microbiol.

[51] Weening EH, Barker JD, Laarakker MC, Humphries AD, Tsolis RM (2005). The *Salmonella enterica* serotype Typhimurium *lpf, bcf, stb, stc, std, and sth* fimbrial operons are required for intestinal persistence in mice.. Infect Immun.

[52] Clayton DJ, Bowen AJ, Hulme SD, Buckley AM, Deacon VL (2008). Analysis of the role of 13 major fimbrial subunits in colonisation of the chicken intestines by *Salmonella enterica* serovar Enteritidis reveals a role for a novel locus.. BMC Microbiol.

[53] Thomson NR, Clayton DJ, Windhorst D, Vernikos G, Davidson S (2008). Comparative genome analysis of *Salmonella* Enteritidis PT4 and *Salmonella* Gallinarum 287/91 provides insights into evolutionary and host adaptation pathways.. Genome Res.

[54] Boddicker JD, Ledeboer NA, Jagnow J, Jones BD, Clegg S (2002). Differential binding to and biofilm formation on, HEp-2 cells by *Salmonella enterica* serovar Typhimurium is dependent upon allelic variation in the fimH gene of the fim gene cluster.. Mol Microbiol.

[55] Kisiela D, Laskowska A, Sapeta A, Kuczkowski M, Wieliczko A (2006). Functional characterization of the FimH adhesin from *Salmonella enterica* serovar Enteritidis.. Microbiology.

[56] Grzymajlo K, Kuzminska-Bajor M, Jaworski J, Dobryszycki P, Ugorski M (2010). The high-adhesive properties of the FimH adhesin of *Salmonella enterica* serovar Enteritidis are determined by a single F118S substitution.. Microbiology.

[57] Guo A, Lasaro MA, Sirard J-C, Kraehenbühl J-P, Schifferli DM (2007). Adhesin-dependent binding and uptake of *Salmonella enterica* serovar Typhimurium by dendritic cells.. Microbiology.

[58] Kisiela D, Sapeta A, Kuczkowski M, Stefaniak T, Wieliczko A (2005). Characterization of FimH adhesins expressed by *Salmonella enterica* serovar Gallinarum biovars Gallinarum and Pullorum: reconstitution of mannose-binding properties by single amino acid substitution.. Infect Immun.

[59] Guo A, Cao S, Tu L, Chen P, Zhang C (2009). FimH alleles direct preferential binding of *Salmonella* to distinct mammalian cells or to avian cells.. Microbiology.

[60] Old DC, Tavendale A, Senior BW (1985). A comparative study of the type-3 fimbriae of *Klebsiella* species.. Journal of medical microbiology.

[61] Schifferli DM, Curtiss III R (2005). Adhesins of enterotoxigenic *Escherichia coli* strains that infect animals..

[62] Chessa D, Winter MG, Jakomin M, Baumler AJ (2009). *Salmonella enterica* serotype Typhimurium Std fimbriae bind terminal alpha(1,2)fucose residues in the cecal mucosa.. Mol Microbiol.

[63] Yen MR, Peabody CR, Partovi SM, Zhai Y, Tseng YH (2002). Protein-translocating outer membrane porins of Gram-negative bacteria.. Biochimica et biophysica acta.

[64] Boyd EF, Wang FS, Whittam TS, Selander RK (1996). Molecular genetic relationships of the salmonellae.. Appl Environ Microbiol.

[65] Seth-Smith HM, Fookes MC, Okoro CK, Baker S, Harris SR (2012). Structure, diversity, and mobility of the Salmonella pathogenicity island 7 family of integrative and conjugative elements within Enterobacteriaceae.. J Bacteriol.

[66] Porwollik S, Wong RM, McClelland M (2002). Evolutionary genomics of *Salmonella*: gene acquisitions revealed by microarray analysis.. Proceedings of the National Academy of Sciences of the United States of America.

[67] Porwollik S, Boyd EF, Choy C, Cheng P, Florea L (2004). Characterization of *Salmonella enterica* subspecies I genovars by use of microarrays.. J Bacteriol.

[68] Porwollik S, McClelland M (2003). Lateral gene transfer in *Salmonella*.. Microbes and infection/Institut Pasteur.

[69] Waack S, Keller O, Asper R, Brodag T, Damm C (2006). Score-based prediction of genomic islands in prokaryotic genomes using hidden Markov models.. BMC bioinformatics.

[70] Fricke WF, Mammel MK, McDermott PF, Tartera C, White DG (2011). Comparative genomics of 28 *Salmonella enterica* isolates: evidence for CRISPR-mediated adaptive sublineage evolution.. Journal of bacteriology.

[71] Lienau EK, Strain E, Wang C, Zheng J, Ottesen AR (2011). Identification of a salmonellosis outbreak by means of molecular sequencing.. N Engl J Med.

[72] Welch TJ, Fricke WF, McDermott PF, White DG, Rosso ML (2007). Multiple antimicrobial resistance in plague: an emerging public health risk.. PLoS One.

[73] Lee JH, Isaacson RE (1995). Expression of the gene cluster associated with the *Escherichia coli* pilus adhesin K99.. Infect Immun.

[74] Edwards RA, Cao J, Schifferli DM (1996). Identification of major and minor chaperone proteins involved in the export of 987P fimbriae.. J Bacteriol.

[75] Clegg S, Wilson J, Johnson J (2011). More than one way to control hair growth: regulatory mechanisms in enterobacteria that affect fimbriae assembled by the chaperone/usher pathway.. Journal of bacteriology.

[76] Benz I, Schmidt MA (2011). Structures and functions of autotransporter proteins in microbial pathogens.. International journal of medical microbiology: IJMM.

[77] Bratlie MS, Johansen J, Sherman BT, Huang da W, Lempicki RA (2010). Gene duplications in prokaryotes can be associated with environmental adaptation.. BMC genomics.

[78] Kugelberg E, Kofoid E, Andersson DI, Lu Y, Mellor J (2010). The tandem inversion duplication in *Salmonella enterica*: selection drives unstable precursors to final mutation types.. Genetics.

[79] Ballouz S, Francis AR, Lan R, Tanaka MM (2010). Conditions for the evolution of gene clusters in bacterial genomes.. PLoS computational biology.

[80] Clarkson LS, Tobin-D’Angelo M, Shuler C, Hanna S, Benson J (2010). Sporadic S*almonella enterica* serotype Javiana infections in Georgia and Tennessee: a hypothesis-generating study.. Epidemiology and infection.

[81] Arthurson V, Sessitsch A, Jaderlund L (2011). Persistence and spread of *Salmonella enterica* serovar Weltevreden in soil and on spinach plants.. FEMS microbiology letters.

[82] Brankatschk K, Blom J, Goesmann A, Smits TH, Duffy B (2011). Genome of a European fresh-vegetable food safety outbreak strain of *Salmonella enterica* subsp. enterica serovar weltevreden.. Journal of bacteriology.

[83] Sheth AN, Hoekstra M, Patel N, Ewald G, Lord C (2011). A national outbreak of *Salmonella* serotype tennessee infections from contaminated peanut butter: a new food vehicle for salmonellosis in the United States.. Clinical infectious diseases : an official publication of the Infectious Diseases Society of America.

[84] Sivapalasingam S, Hoekstra RM, McQuiston JR, Fields PI, Tauxe RV (2004). *Salmonella* bacteriuria: an increasing entity in elderly women in the United States.. Epidemiology and infection.

[85] Haahtela K, Laakso T, Korhonen TK (1986). Associative Nitrogen Fixation by *Klebsiella* spp.: Adhesion Sites and Inoculation Effects on Grass Roots.. Applied and environmental microbiology.

[86] Francis DH, Erickson AK, Grange PA (1999). K88 adhesins of enterotoxigenic *Escherichia coli* and their porcine enterocyte receptors.. Adv Exp Med Biol.

[87] Bäumler AJ, Tsolis RM, Heffron F (1996). The *lpf* fimbrial operon mediates adhesion of *Salmonella typhimurium* to murine Peyer’s patches.. Proc Natl Acad Sci USA.

[88] Holt KE, Thomson NR, Wain J, Langridge GC, Hasan R (2009). Pseudogene accumulation in the evolutionary histories of *Salmonella enterica* serovars Paratyphi A and Typhi.. BMC genomics.

[89] Chiu CH, Tang P, Chu C, Hu S, Bao Q (2005). The genome sequence of *Salmonella enterica* serovar Choleraesuis, a highly invasive and resistant zoonotic pathogen.. Nucleic acids research.

[90] Liu WQ, Feng Y, Wang Y, Zou QH, Chen F (2009). *Salmonella* paratyphi C: genetic divergence from *Salmonella* choleraesuis and pathogenic convergence with *Salmonella* typhi.. PloS one.

[91] Bolton AJ, Martin GD, Osborne MP, Wallis TS, Stephen J (1999). Invasiveness of *Salmonella* serotypes Typhimurium, Choleraesuis and Dublin for rabbit terminal ileum in vitro.. Journal of medical microbiology.

[92] Berger CN, Shaw RK, Brown DJ, Mather H, Clare S (2009). Interaction of *Salmonella enterica* with basil and other salad leaves.. The ISME journal.

[93] Berger CN, Sodha SV, Shaw RK, Griffin PM, Pink D (2010). Fresh fruit and vegetables as vehicles for the transmission of human pathogens.. Environmental microbiology.

[94] Golberg D, Kroupitski Y, Belausov E, Pinto R, Sela S (2011). *Salmonella* Typhimurium internalization is variable in leafy vegetables and fresh herbs.. International journal of food microbiology.

[95] Tsolis RM, Townsend SM, Miao EA, Miller SI, Ficht TA (1999). Identification of a putative *Salmonella enterica* serotype typhimurium host range factor with homology to IpaH and YopM by signature-tagged mutagenesis.. Infect Immun.

[96] Forest C, Faucher SP, Poirier K, Houle S, Dozois CM (2007). Contribution of the *stg* fimbrial operon of *Salmonella enterica* serovar Typhi during interaction with human cells.. Infect Immun.

[97] Hase K, Kawano K, Nochi T, Pontes GS, Fukuda S (2009). Uptake through glycoprotein 2 of FimH(+) bacteria by M cells initiates mucosal immune response.. Nature.

[98] Kisiela DI, Kramer JJ, Tchesnokova V, Aprikian P, Yarov-Yarovoy V (2011). Allosteric catch bond properties of the FimH adhesin from *Salmonella enterica* serovar Typhimurium.. The Journal of biological chemistry.

[99] Liu F, Kariyawasam S, Jayarao BM, Barrangou R, Gerner-Smidt P (2011). Subtyping *Salmonella enterica* serovar enteritidis isolates from different sources by using sequence typing based on virulence genes and clustered regularly interspaced short palindromic repeats (CRISPRs).. Applied and environmental microbiology.

[100] Liu F, Barrangou R, Gerner-Smidt P, Ribot EM, Knabel SJ (2011). Novel virulence gene and clustered regularly interspaced short palindromic repeat (CRISPR) multilocus sequence typing scheme for subtyping of the major serovars of *Salmonella enterica* subsp. *enterica*.. Applied and environmental microbiology.

[101] Dwyer BE, Newton KL, Kisiela D, Sokurenko EV, Clegg S (2011). Single nucleotide polypmorphisms of *fimH* associated with adherence and biofilm formation by serovars of *Salmonella enterica*.. Microbiology.

[102] Sokurenko EV, Vogel V, Thomas WE (2008). Catch-bond mechanism of force-enhanced adhesion: counterintuitive, elusive, but … widespread?. Cell Host Microbe.

[103] Tchesnokova V, McVeigh AL, Kidd B, Yakovenko O, Thomas WE (2010). Shear-enhanced binding of intestinal colonization factor antigen I of enterotoxigenic *Escherichia coli*.. Molecular microbiology.

[104] Stahlhut SG, Tchesnokova V, Struve C, Weissman SJ, Chattopadhyay S (2009). Comparative structure-function analysis of mannose-specific FimH adhesins from *Klebsiella pneumoniae* and *Escherichia coli*.. Journal of bacteriology.

[105] Tchesnokova V, Aprikian P, Kisiela D, Gowey S, Korotkova N (2011). Type 1 Fimbrial Adhesin FimH Elicits an Immune Response That Enhances Cell Adhesion of *Escherichia coli*.. Infection and immunity.

[106] Beaver JE, Bourne PE, Ponomarenko JV (2007). EpitopeViewer: a Java application for the visualization and analysis of immune epitopes in the Immune Epitope Database and Analysis Resource (IEDB).. Immunome research.

[107] Maurelli AT (2007). Black holes, antivirulence genes, and gene inactivation in the evolution of bacterial pathogens.. FEMS microbiology letters.

[108] Soyer Y, Orsi RH, Rodriguez-Rivera LD, Sun Q, Wiedmann M (2009). Genome wide evolutionary analyses reveal serotype specific patterns of positive selection in selected *Salmonella* serotypes.. BMC evolutionary biology.

[109] Aziz RK, Bartels D, Best AA, DeJongh M, Disz T (2008). The RAST Server: rapid annotations using subsystems technology.. BMC Genomics.

[110] Altschul SF, Madden TL, Schaffer AA, Zhang J, Zhang Z (1997). Gapped BLAST and PSI-BLAST: a new generation of protein database search programs.. Nucleic Acids Res.

[111] Choudhury D, Thompson A, Stojanoff V, Langermann S, Pinkner J (1999). X-ray structure of the FimC-FimH chaperone-adhesin complex from uropathogenic *Escherichia coli*.. Science.

[112] Li YF, Poole S, Rasulova F, McVeigh AL, Savarino SJ (2007). A receptor-binding site as revealed by the crystal structure of CfaE, the colonization factor antigen I fimbrial adhesin of enterotoxigenic *Escherichia coli*.. The Journal of biological chemistry.

[113] Dodson KW, Pinkner JS, Rose T, Magnusson G, Hultgren SJ (2001). Structural basis of the interaction of the pyelonephritic *E. coli* adhesin to its human kidney receptor.. Cell.

[114] Tamura K, Peterson D, Peterson N, Stecher G, Nei M (2011). MEGA5: Molecular Evolutionary Genetics Analysis using Maximum Likelihood, Evolutionary Distance, and Maximum Parsimony Methods.. Molecular biology and evolution.

[115] Kelley LA, Sternberg MJ (2009). Protein structure prediction on the Web: a case study using the Phyre server.. Nature protocols.

[116] McGuffin LJ (2007). Benchmarking consensus model quality assessment for protein fold recognition.. BMC bioinformatics.

[117] Wu S, Zhang Y (2008). MUSTER: Improving protein sequence profile-profile alignments by using multiple sources of structure information.. Proteins.

[118] Roy A, Kucukural A, Zhang Y (2010). I-TASSER: a unified platform for automated protein structure and function prediction.. Nature protocols.

[119] Hung CS, Bouckaert J, Hung D, Pinkner J, Widberg C (2002). Structural basis of tropism of *Escherichia coli* to the bladder during urinary tract infection.. Mol Microbiol.

[120] Garcia-Boronat M, Diez-Rivero CM, Reinherz EL, Reche PA (2008). PVS: a web server for protein sequence variability analysis tuned to facilitate conserved epitope discovery.. Nucleic acids research.

[121] Kinoshita K, Nakamura H (2003). Identification of protein biochemical functions by similarity search using the molecular surface database eF-site.. Protein science : a publication of the Protein Society.

[122] Delport W, Poon AF, Frost SD, Kosakovsky Pond SL (2010). Datamonkey 2010: a suite of phylogenetic analysis tools for evolutionary biology.. Bioinformatics.

