# Supplementary figures and images for: Diversification of the Salmonella Fimbriae: A Model of Macro- and Microevolution
Source: PLoS One. 2012 Jun 12;7(6):e38596. doi: 10.1371/journal.pone.0038596 (PMC3373541; doi:10.1371/journal.pone.0038596)

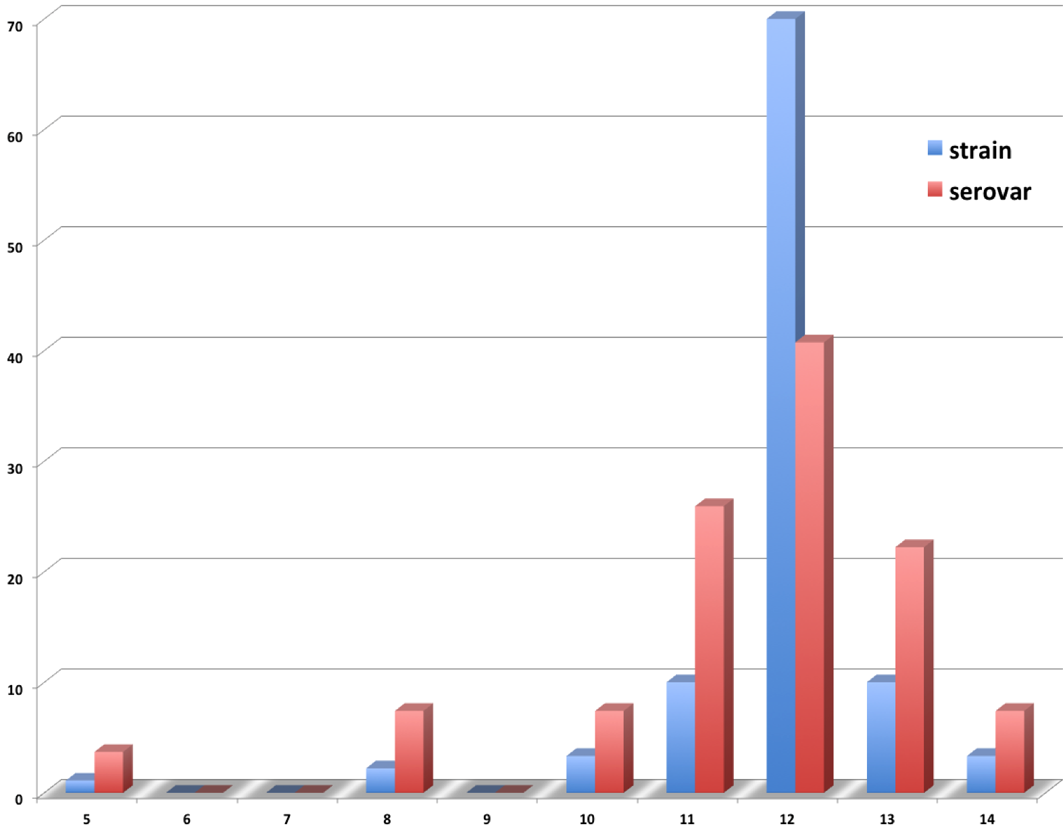

Supplement: Figure S1 — Relative numbers of FGCs per strain or per serovar. The horizontal axis shows the numbers of fimbriae per strain, and the vertical axis shows the corresponding percentages of strains (blue) or serovars (red) for each number. (TIF) [file pone.0038596.s001.tif]

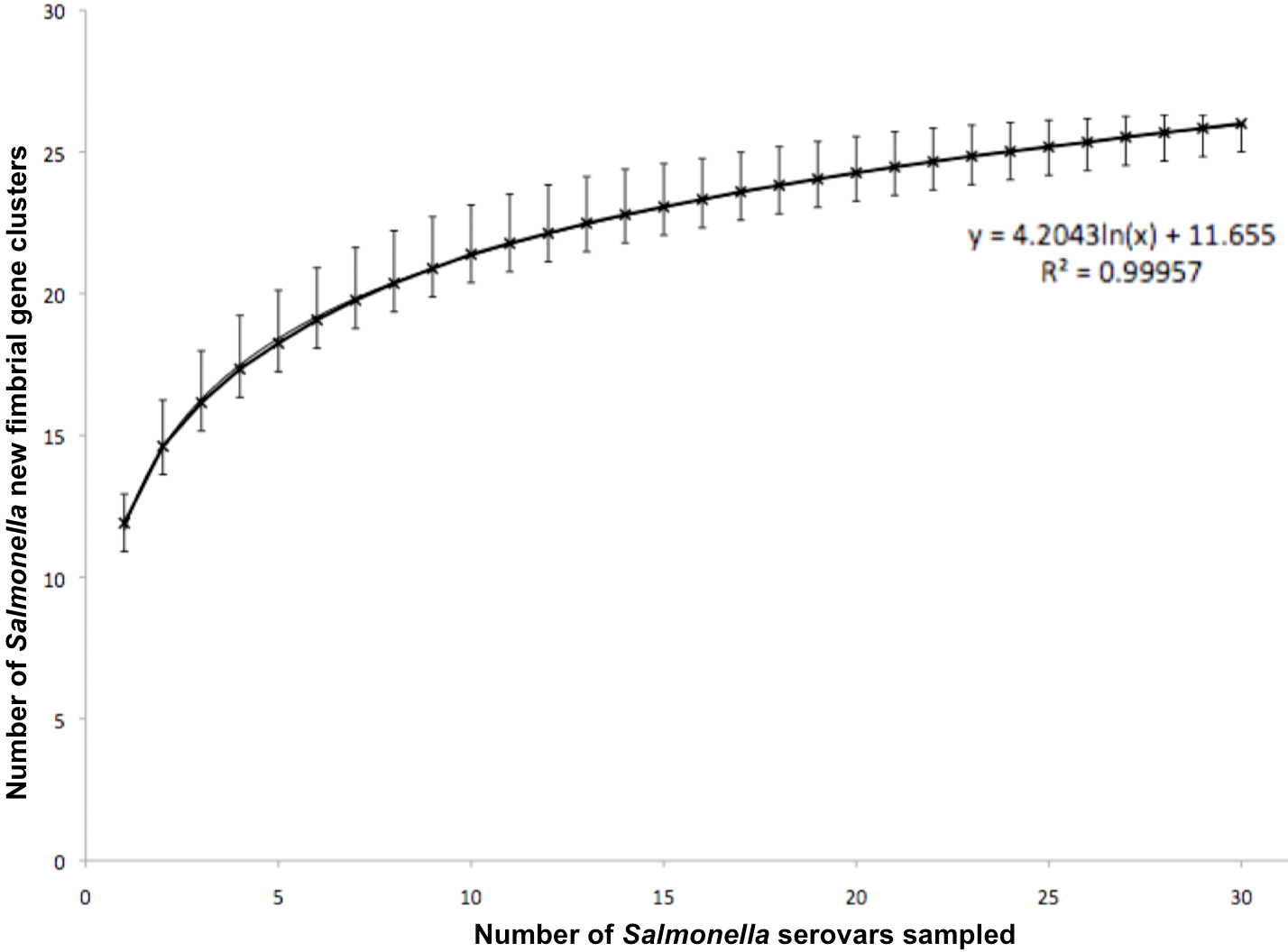

Supplement: Figure S2 — Collector’s curve for the prediction of the total number of different FGCs in S. enterica subsp. I. The 26 FGCs of serovars Dublin, Gallinarum, Newport, Saintpaul and Typhimurium were used to construct the curve. The obtained curve was fitted to a logarithmic curve with the equation y = 4.2043 ln(×) +11.655 (r2 = 0.99957). (TIF) [file pone.0038596.s002.tif]

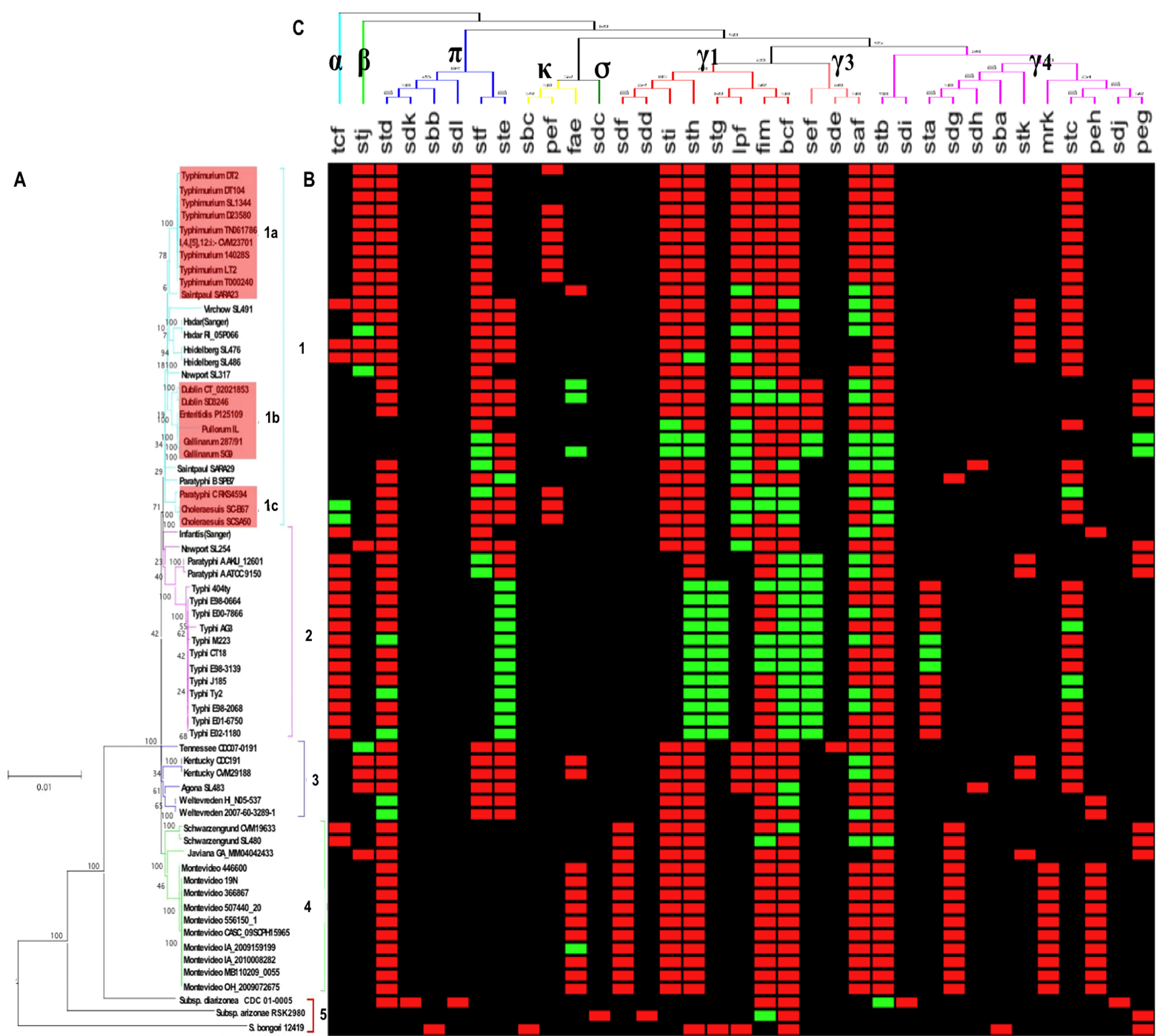

Supplement: Figure S3 — Evolution of Salmonella along with their FGCs. On the left, phylogenomic tree of 90 Salmonella derived from 45 highly conserved house-keeping genes (see Methods) totaling ∼43 Kb. All the Salmonella formed five distinct clades labeled 1 to 5 (as shown in Figures 2 and 3). The scale indicates the number of substitutions per nucleotide. Heat map, distribution of FGCs; FGCs with or without pseudogenes were shown as green or red rectangles, respectively. On the top, phylogenetic tree for the FGCs, adopted from Figure 1. (TIF) [file pone.0038596.s003.tif]

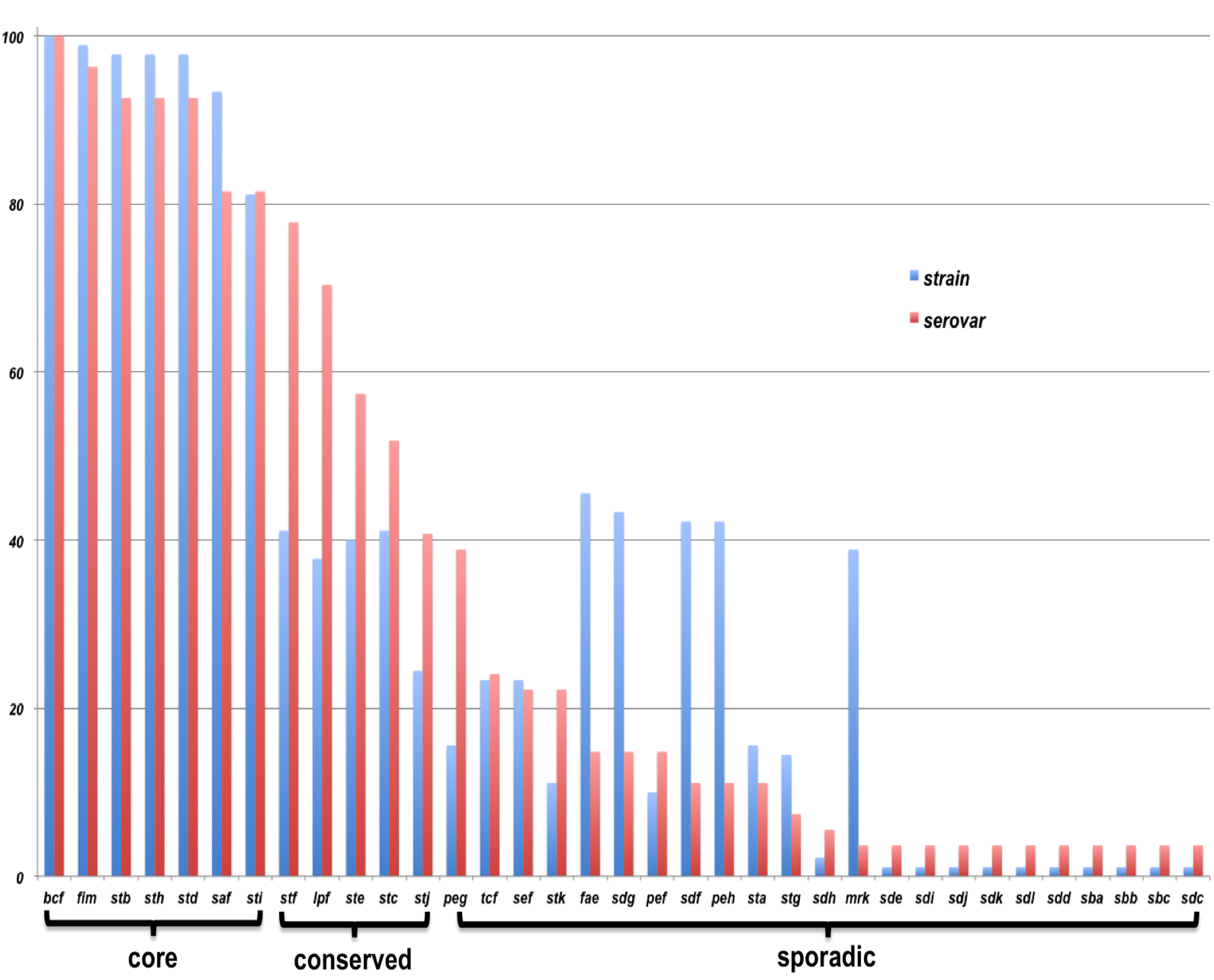

Supplement: Figure S4 — Relative frequency distribution for each FGC. The horizontal axis shows each of the 35 distinct FGCs and the vertical axis shows corresponding percentages of strains (blue) or serovars (red) for each FGCs. The data were from 90 strains covering 27 serovars. (TIF) [file pone.0038596.s004.tif]

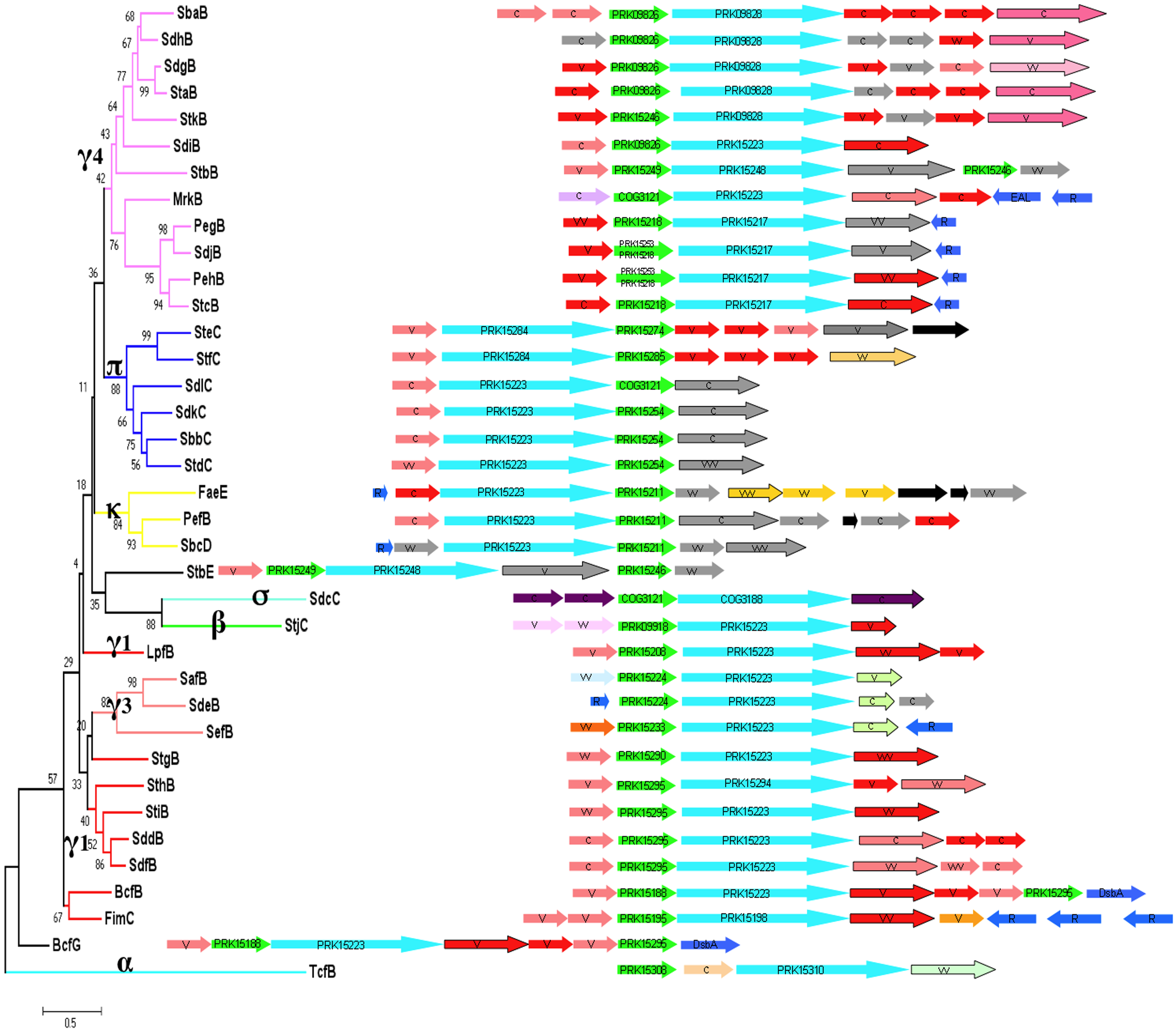

Supplement: Figure S5 — Phylogenic tree for Salmonella fimbrial chaperones. A phylogenetic tree was built for all identified FGCs in the Salmonella pangenome by using the amino acid sequences of the combined 1094 chaperone proteins (MEGA 5.0, as described in method). The FGCs were divided into five clades and the genes were color-coded as shown in Figure 1. The scale indicates the number of substitutions per amino acid. (TIF) [file pone.0038596.s005.tif]

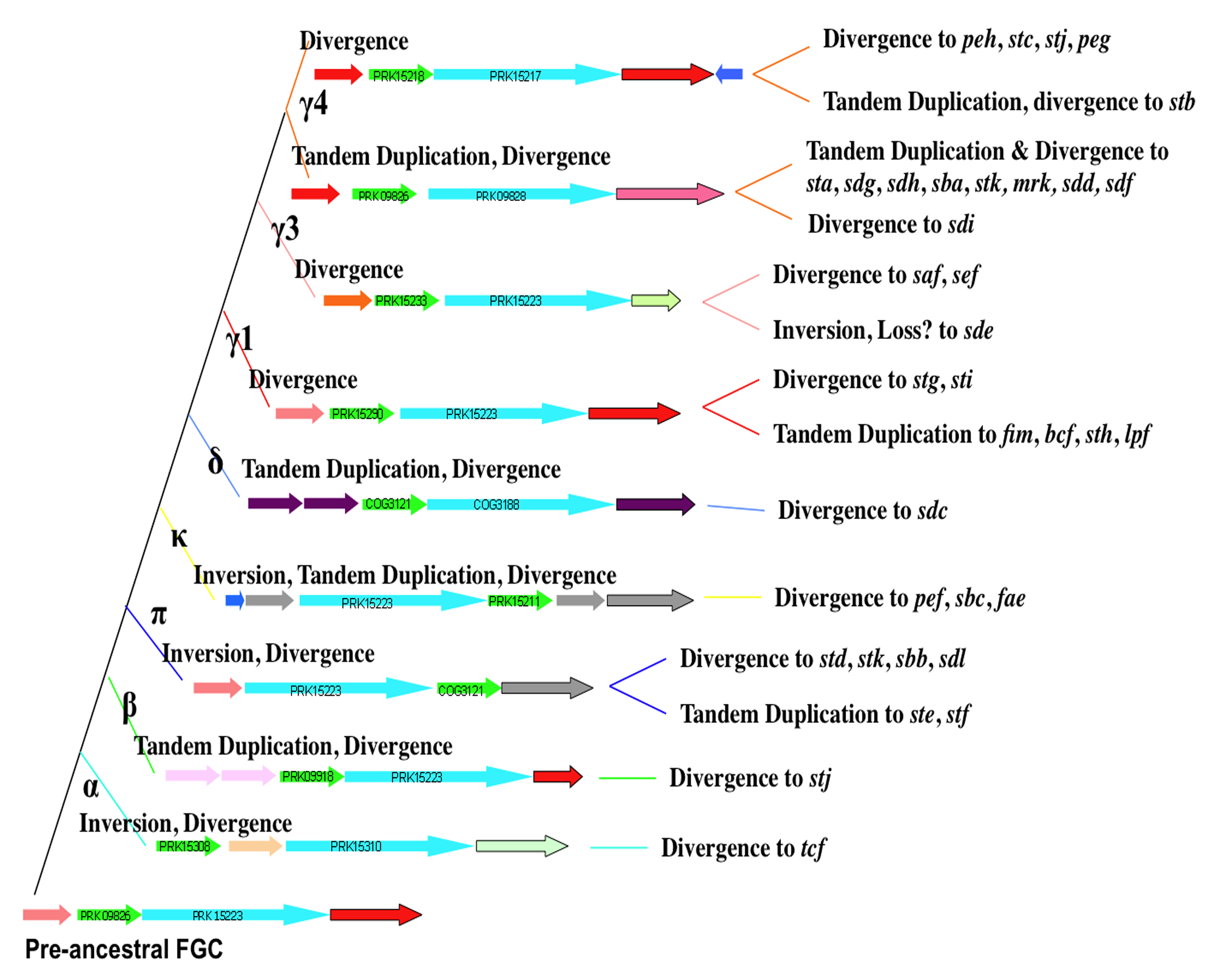

Supplement: Figure S6 — Evolution model for the Salmonella fimbriome. The proposed evolution pathway (and color code) is based on the FGC classification shown in Figure 1 and starts at the bottom of the figure with a prototypical ancestral FGC. (TIF) [file pone.0038596.s006.tif]

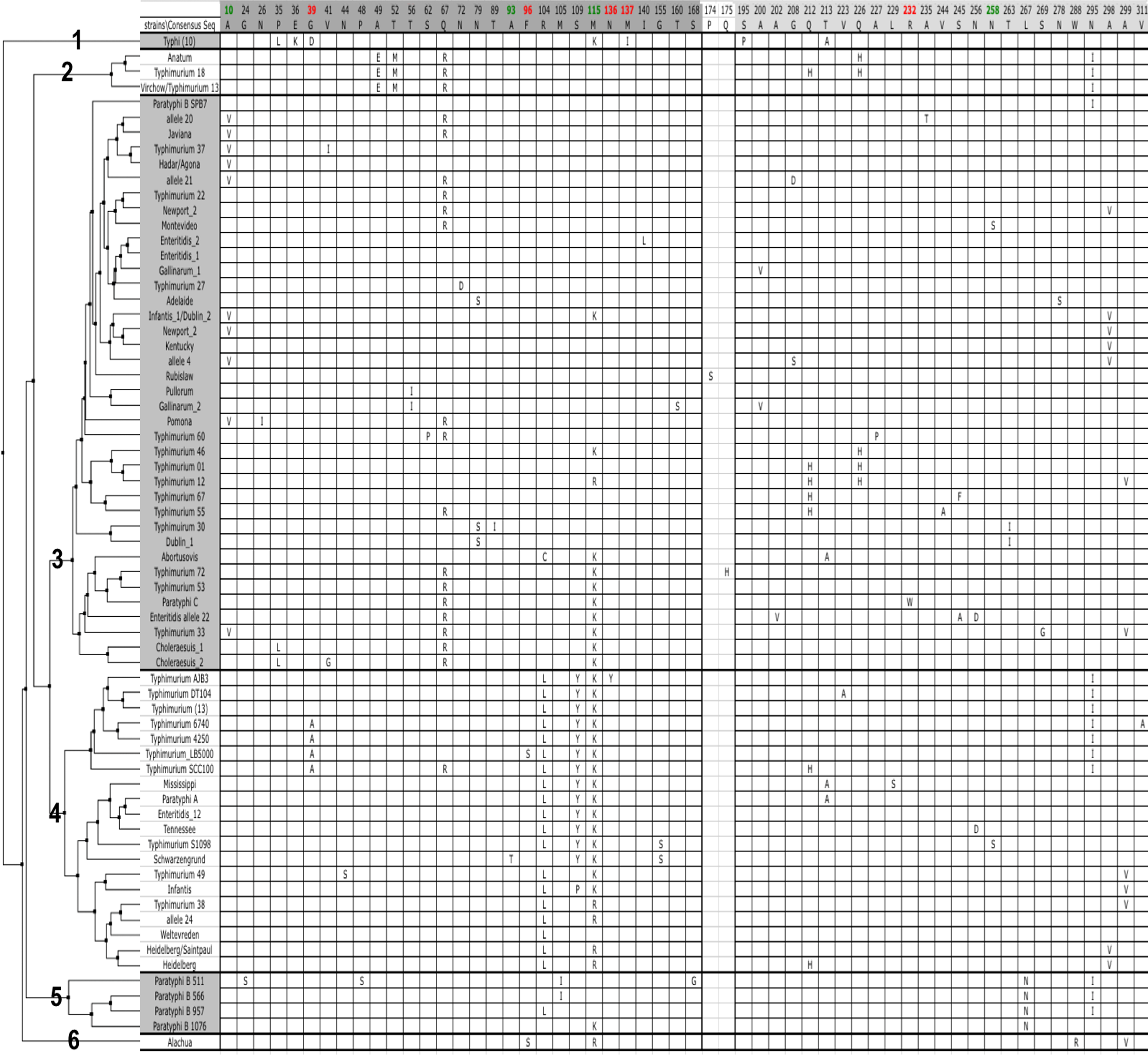

Supplement: Figure S7 — FimH alleles in Salmonella subspecies I. Available genomic and protein sequence data identified 67 FimH alleles in Salmonella subspecies I. The alleles were divided in six groups (1 to 6, on the left) based on a FimH average distance tree produced by using BLOSUM62. The top two rows list the substituted FimH residue positions with the most prevalent residue at this position. The background for the lectin domain residues is labeled in dark gray, whereas the background of the pili domain residues is labeled in light gray (linker domain in white). Residue positions in red correspond to mannose-binding enhancing substitutions and residue positions in green correspond to mannose-binding neutral substitutions; residue substitution effects on mannose binding in other positions is not known. (TIF) [file pone.0038596.s007.tif]
